# Smart Polymer Surfaces with Complex Wrinkled Patterns: Reversible, Non-Planar, Gradient, and Hierarchical Structures

**DOI:** 10.3390/polym15030612

**Published:** 2023-01-25

**Authors:** Mauricio A. Sarabia-Vallejos, Felipe E. Cerda-Iglesias, Dan A. Pérez-Monje, Nicolas F. Acuña-Ruiz, Claudio A. Terraza-Inostroza, Juan Rodríguez-Hernández, Carmen M. González-Henríquez

**Affiliations:** 1 Facultad de Ingeniería, Arquitectura y Diseño, Universidad San Sebastián, Sede Santiago, Santiago 8420524, Chile; 2 Departamento de Química, Facultad de Ciencias Naturales, Matemáticas y del Medio Ambiente, Universidad Tecnológica Metropolitana, Santiago 7800003, Chile; 3 Programa PhD en Ciencia de Materiales e Ingeniería de Procesos, Universidad Tecnológica Metropolitana, Santiago 8940000, Chile; 4 Research Laboratory for Organic Polymer (RLOP), Facultad de Química y Farmacia, Pontificia Universidad Católica de Chile, Santiago 7810000, Chile; 5 Polymer Functionalization Group, Departamento de Química Macromolecular Aplicada, Instituto de Ciencia y Tecnología de Polímeros-Consejo Superior de Investigaciones Científicas (ICTP-CSIC), 28006 Madrid, Spain; 6 Programa Institucional de Fomento a la Investigación, Desarrollo e Innovación, Universidad Tecnológica Metropolitana, Santiago 8940000, Chile

**Keywords:** wrinkles surface, surface microstructuration, smart interfaces, non-planar structuration, hierarchical surface structures

## Abstract

This review summarizes the relevant developments in preparing wrinkled structures with variable characteristics. These include the formation of smart interfaces with reversible wrinkle formation, the construction of wrinkles in non-planar supports, or, more interestingly, the development of complex hierarchically structured wrinkled patterns. Smart wrinkled surfaces obtained using light-responsive, pH-responsive, temperature-responsive, and electromagnetic-responsive polymers are thoroughly described. These systems control the formation of wrinkles in particular surface positions and the reversible construction of planar-wrinkled surfaces. This know-how of non-planar substrates has been recently extended to other structures, thus forming wrinkled patterns on solid, hollow spheres, cylinders, and cylindrical tubes. Finally, this bibliographic analysis also presents some illustrative examples of the potential of wrinkle formation to create more complex patterns, including gradient structures and hierarchically multiscale-ordered wrinkles. The orientation and the wrinkle characteristics (amplitude and period) can also be modulated according to the requested application.

## 1. Introduction

The design, development, and testing of stimuli-responsive materials are critical for several application areas. Thus, the dynamic and reversible control of surface topography has been extensively studied due to their use, such as smart optics and diffraction grating for tunable light manipulation [1], wearable tactile devices, micropatterns to control friction between solid surfaces separated by a thin fluid film [2,3], refreshable Braille surfaces [4,5], anti-biofouling coating [6], adjustable wettability [7], smart adhesion [8], e-skin and stretchable devices [9,10]. Compared with traditional static surfaces, these microstructured responsive surfaces are highly desired due to their capacity to tune responses, creating more flexible devices suitable for soft electronic and haptic engineering. These applications need the materials to actively change their characteristics, geometry, or function to fulfill each case’s requirements. Polymers have been widely used to fabricate these devices because they present several advantages against other materials (ceramic, alloys, or hybrids), like easy and low-cost fabrication; besides, polymers are simple to employ. In general, their properties can be adjusted using a straightforward synthetic methodology. Also, various stimuli-responsive polymers are biocompatible or biodegradable, making them suitable for several biomedical applications [11,12].

### 1.1. Smart Polymers

Smart materials have intrinsic properties that allow them to experiment with physical or chemical changes in response to external stimuli, including slight variations in the surrounding environment, such as temperature, light, electric/magnetic fields, mechanical stress, pH, humidity, ionic strength and existence of metabolic chemicals [13].

According to Jingcheng et al. [14], in the last two decades (since 2001), there have been significant progress and publications in the area of smart polymers, increasing from 4000 articles published in 2001 to more than 14,000 in the year 2020. The first scientist to coin the name “smart polymers” was Dagani et al. in 1995, who highlighted the similarity of these materials with some biopolymers that present “responsive” natures. Other authors compare smart polymers with machines, which can convert an energy input into mechanical work (shape-shifting). The decisive benefit of smart materials for medical device design is the simplicity of the resulting devices compared to complex mechanical engineering solutions [15].

Smart polymers could change their structure at different length scales ranging from macro- to micrometer sizes. These morphological changes or transitions are usually reversible and return to their initial state when the external stimulus is removed. Recent studies demonstrated that these compounds could change their shape and other characteristics like solubility or modify properties such as wettability or adhesion when placed on surfaces. Similarly, sol-gel transitions or molecular self-assemblies can occur spontaneously on these materials. From the macroscopic level, the adaptive performances of smart materials are achieved by the autonomous behaviors of molecules or atoms at a microscopic or nanoscopic level. When stimuli are introduced, aggregation, rearrangement, and directional movement of the molecules/atoms can occur, thus triggering different reactions in the macroscopical behavior of the material [14].

According to Raquez and Peponi [16], there are two main types of smart materials: self-evolving and self-healing. The first can, for example, reconfigure themselves “on the fly” to react to changing environmental conditions. Some cases of these kinds of materials are shape-memory polymers, adaptive hydrogels, and actuators. In addition, intrinsic self-healing polymers are synthesized on reversible bonding, such as Diels-Alder reactions.

Zhou et al. [17] described a different categorization for self-evolving materials, which can be subdivided into four groups: self-assembly [18], deformation mismatch [19], bi-stability, and shape-memory effect [20,21,22]. This last category can be classified into five different families, where shape-memory polymers (SMPs) and shape-memory alloys (SMAs) [22,23,24] are the two most important.

In summary, the unique features of smart polymers enable their use in different technological areas, for example, for biomedical purposes such as drug delivery systems [25] or for being used in cardiovascular implants [15], or as part of microscale machines [26] such as devices for data storage, smart textiles, or robotic e-skin.

### 1.2. Instability-Based Surface Patterning: Formation of Wrinkled Polymer Surfaces

The observation of wrinkles on polymer surfaces has been widely studied. Thus, the first reports evidencing the formation of wrinkled coatings using China wood oil treated at high temperatures under atmospheric oxygen. During World War II, airplane wings were fabricated using crosslinked polymer foams and rigid composites, which, under bendings and compressions, could generate these wrinkled patterns on the surface. Despite the initial objection to using these coatings, their use was extended, giving to wrinkled finished coatings [27].

In the last two decades, the interest in this type of surface has increased considerably, being the study of Mahadevan et al. [28] and Genzer et al. [29], the ones who kicked off the renaissance of microstructured wrinkled surfaces. Mahadevan et al. set the physical foundations surrounding the formation of these patterns and explained how the accumulation of tension in the film triggers the formation of ordered wrinkles patterns on the surface of the material. On the other hand, Genzer and their group analyze the processes involved in forming nested wrinkled patterns using elastomeric substrates, extending its dimensions over five orders of magnitude (from a few nanometers to millimeters). Their experimental results were contrasted with both computations and a simple scaling theory, demonstrating that it is possible to predict and model the wrinkling pattern formation. These studies are considered the cornerstone of recent methodologies used to create wrinkled patterns, which implies that none of the studies, applications and technologies reviewed in this article would be possible without these two articles.

Recently, several material scientists have been focused on reproducing some of the properties or behaviors that natural/biological materials present. For instance, biomimetic materials are used repeatedly in tissue engineering (TE) [30] to fabricate pieces or devices that resemble natural tissues or organs. These materials can alter their designs by stimulating specific cellular responses associated with new tissue formation mediated by biomolecular recognition.

The great effort carried out in elaborating these materials is justified by the wide variety of applications that largely depend on the surface properties of a particular material. For instance, both patterning and functionalization of polymer surfaces have been demonstrated to play a crucial role in the preparation of substrates for different applications in the biomedical [30], soft electronic [31], optical [32], or micromechanical [33] areas, among others.

In this context, many of the strategies reported to reproduce the superior properties of surfaces in nature failed or resorted to intricate methodologies that require the use of expensive and sophisticated equipment. In this sense, some advances in the development of polymeric surfaces have been associated with technological improvements.

However, more recently, several alternative approaches have been developed in which the origin of surface patterns is not the result of advanced fabrication techniques but takes advantage of the instabilities produced in these polymeric surfaces [34]. Two alternative routes can be used to create surface instabilities; firstly, they could be fabricated spontaneously in small-scale compact systems, which are particularly unstable. On the other hand, a specific external stimulus can induce a surface fluctuation in metastable films. These stimuli include mechanical force (stretching or compression), electric field, or heating [27]. After removal, relaxation of the surface towards equilibrium results in modification of the film structure to minimize the free energy in the film. It can be seen that different nano and micrometric size morphologies with particular morphologies and distributions are generated on the surface of the material. In particular, the latter allows the formation of intricate patterns, such as wrinkle or fold patterns, which would be complicated, if not impossible, to fabricate by conventional micro-modification methods.

**Figure 1:**
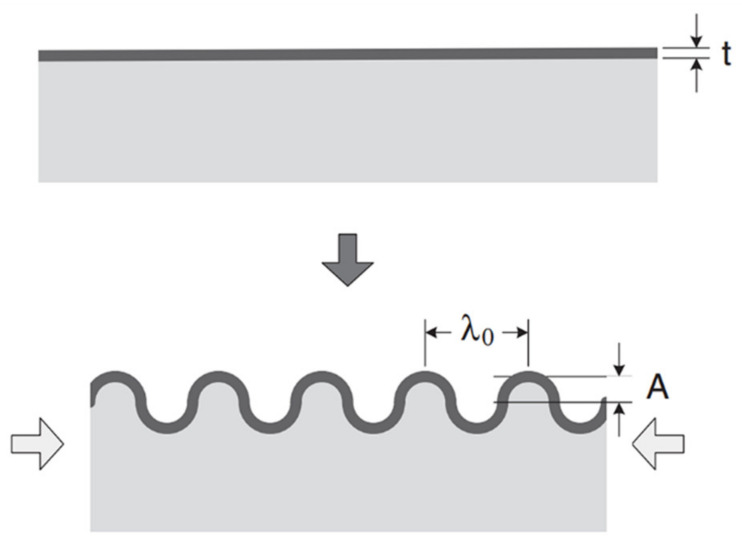
Illustration of the general mechanisms for forming wrinkles in bilayered systems comprising a rigid top layer on an elastic substrate. Reproduced with permission from ref. [35]. Copyright 2010, John Wiley & Sons, Inc.

Accordingly, the general wrinkling system based on surface instabilities is illustrated in Figure 1. For the case of the bilayer system consisting of a rigid top layer over an elastic layer, the first step requires the application of a mechanical tensile force (originating from osmotic pressure, stretching, or heating, to name a few). Next, the rigid layer is deposited on the polymer film. Finally, after the removal of the applied stimulus, the initial shape of the elastic layer relaxes, and a wavy structure identified as “wrinkles” is generated on the surface [35].

This behavior can be explained by considering the soft substrate as an elastic material. The external energy input is partially converted when expanding into elastic potential energy within the substrate. After coating it with a thin hard layer over the soft substrate, followed by the relaxation of the initial stimulus, a new equilibrium state must be relocated due to a mismatch of two equilibrium states between the soft elastomeric substrate and the hard elastic layer [27]. The wrinkle wavelength and amplitude result from the minimization of the total elastic energy in the thin layer and the soft substrate. The wrinkling process relaxes the compressive strain, thus reducing the inner film energy. At the same time, it generates the film’s bending, increasing the material’s elastic strain energy [35].

The wavelength λ_0_ depends on the thickness of the thin hard film (t) and the mechanical properties of the thin film and the substrate:

(1) $$\lambda_{0}=2\pi t{\left(\frac{{\bar{E}}_{f}}{3{\bar{E}}_{s}}\right)}^{1/3}$$

where *Ē* is the plane-strain modulus, defined as E/(1 − ν^2^), with E as Young’s modulus and ν as the Poisson ratio, the subscripts *f* and *s* refer to the top film and the bottom substrates, respectively. It is essential to highlight that the wavelength is independent of the applied strain and stress. On the contrary, the amplitude of the wrinkle pattern depends on the film thickness and the applied strain.

**Figure 2:**
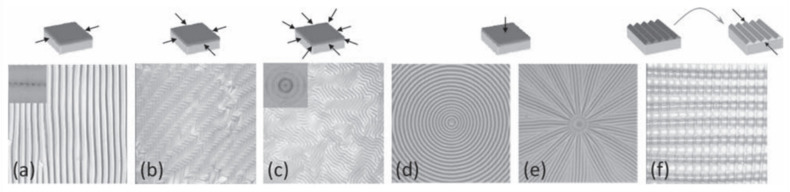
Optical images of various morphological patterns demonstrate that the wrinkling patterns prefer to orient themselves perpendicular to the axis of principal compressive stress: (**a**) stripes (uniaxial mechanical compression), (**b**) herringbones (biaxial mechanical compression), (**c**) labyrinths (isotropic thermal contraction), (**d**) circular, (**e**) radial, and (**f**) checkerboards (uniaxial mechanical compression on a pre-patterned substrate). Reproduced with permission from ref. [37]. Copyright 2011, John Wiley & Sons, Inc.

Different micrometric structures could be formed depending on the dimensions of undulated patterns obtained, known as the aspect ratio of the wrinkles (amplitude/wavelength). These structures could be defined as wrinkles (aspect ratio close to 1), ripples (aspect ratio close to 0), or crumples/folds (aspect ratio higher than 1). The mechanisms of how these structures are formed depend mainly on the material’s mechanical properties and the rigid layer thickness [36]. Similarly, the type of 2D pattern formed on top of the material depends exclusively on the relaxation/compression directions applied to the systems. Figure 2 shows different 2D patterns (lamellar/stripes, herringbone, labyrinths, circular, radial, checkerboards, etc.) that can be formed just by varying the direction of the applied stress [37]. Mixing these micrometric structures (wrinkled patterns) with the surface responsiveness imparted by the smart polymers could resort in an interesting synergy that should improve the surface performance of the materials.

## 2. Desing of Adaptive Wrinkled Patterned Surfaces: Reversible Stimuli-Responsive Wrinkle Formation

As mentioned in the previous paragraphs, surface modification has proven relevant for different applications. Within these, homogeneous and reproducible dynamic surface patterns allow the control of encoded surface functions and properties, generating a convenient or stable method to achieve smart surfaces for different types of devices, whether in various scientific areas, such as optical, smart electronics, sensitive microfluidics, switchable wettability or tunable adhesion [38]. However, the fabrication of dynamic surface patterns remains a major challenge, highlighting the various approaches to fabricating surface patterns with different morphologies. The wrinkle phenomenon is an attractive alternative to generate dynamic patterns due to its spontaneous formation, versatility, easy preparation, scaling power, large area production, and sensitivity to different stimuli [39].

The interactions between the material and the stimuli are responsible for the smart behaviors of the material, which commonly occur in the interface between the material and the environment, so increasing this available surface area increases the device’s smart capabilities. This last property is why wrinkling morphologies are desirable for fabricating smart devices based on polymeric compounds.

The following section discusses some of the most common materials and methodologies used to fabricate wrinkled patterns with reversible stimuli-responsive behavior. Thus, according to the type of stimulus used, these materials were organized in different topics like light-responsive (UV, visible, and NIR), pH-responsive, temperature-responsive, electro-magnetical responsive, mechanical (stress/stretch) responsive, and chemical reaction/gas/solvent responsive.

### 2.1. Light-Responsive Wrinkled Patterned Surfaces

The most used methodologies to fabricate spontaneous wrinkled patterns in the materials’ surface involve light exposure from UV, visible, or NIR sources to induce surface modification.

**Figure 3:**
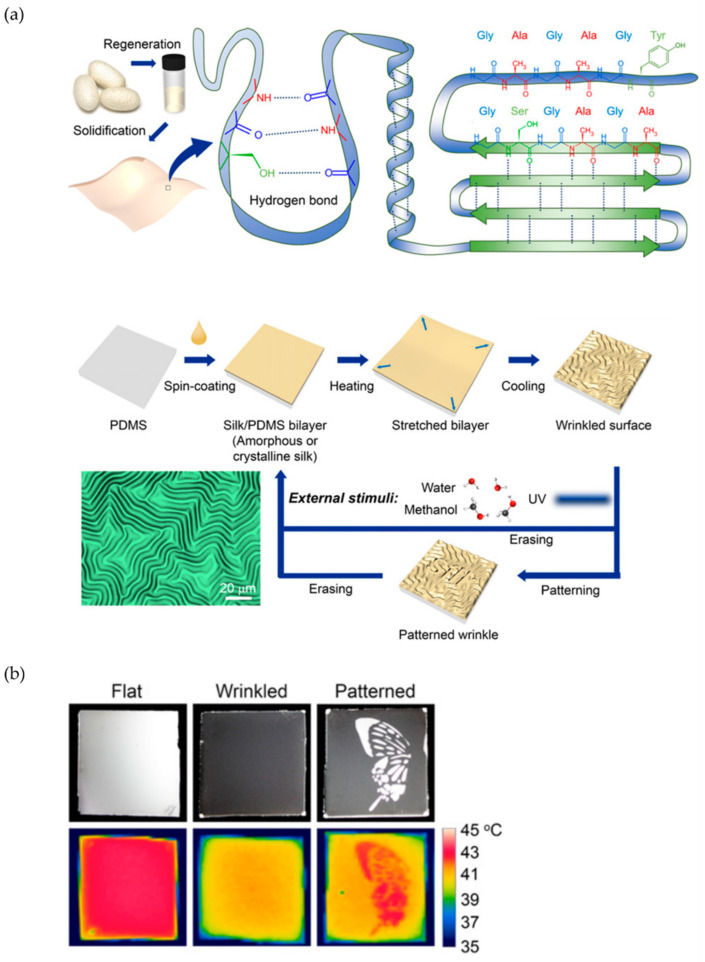
(**a**) Schematic representation of the reconstituted silk-fibroin molecular structure and schematic representation of the process to fabricate the reversible wrinkled patterns. (**b**) Photographs (top) and IR images (bottom) of the flat surface (left), surface with wrinkled structure (middle), and surface with a wrinkled pattern (right). Reproduced with permission from reference [40]. Copyright 2019, Proceedings of the National Academy of Sciences (PNAS).

Several examples of this strategy have been recently reported. For example, Omenneto et al. [40] presented regenerated silk fibroin to explore wrinkle formation by exploiting silk structure-function relation. More precisely, a bilayer system composed of silk/PDMS (polydimethylsiloxane) was fabricated, depositing the silk fibroin solution onto the PDMS substrates by spin-coating. Afterward, the bilayer samples were heated (and therefore stretched) at 140 °C for 5 min and then cooled to room temperature, thus triggering the formation of wrinkling patterned surfaces. In consequence, the protein conformational changes responsible for these surface changes can be induced by exposure to methanol vapor (MV), water vapor (WV), and UV light irradiation (Figure 3). Upon exposure to MV, the stress release behavior is quite fast, thanks to the rapid penetration of the small water molecules into all amorphous regions of the silk matrix of the composite. On the other hand, the mixture of methanol and water molecules penetrates the non-crystalline areas to induce a local molecular rearrangement without significant conformational transition.

In conclusion, the polymorphic transitions of silk fibroin allow orienting the wrinkled patterns by directly influencing the material’s physical properties. The interaction between the silk protein chains and external stimuli controls the protein film’s wrinkling. The silk/PDMS bilayer is mechanically firm and allows reversible and repeatable modification in its wrinkled and unwrinkled states in multiple cycles (more than 50 cycles). Figure 3b shows a possible application in which patterns can be revealed under IR imaging, offering the possibility of displaying IR information.

Jiang et al. provide an effective procedure for generating multi-responsive wrinkles based on a supramolecular polymeric network exposed to UV light. The polymeric network consisted of a pyridine-containing copolymer (P4VP-PS-PnBA) and an anthracene-containing carboxylic acid (AN-COOH) dynamically crosslinked by reversible photodimerization of anthracene (AN) and hydrogen bonding between the carboxyl and pyridine groups. The wrinkling pattern was selectively realized and erased by exposure to acid/base gases and light [41]. Cao, Jiang, and Lu et al. [42] have presented a method to control the formation and removal of dynamically wrinkled patterns by visible light exposure, which is generated by an external stimulus on an azo-containing poly(disperse orange 3) (PDO_3_) film bonded to a PDMS substrate. Light-induced photoisomerization of the azobenzene units in the azo-polymer films triggers the release of tension, resulting in the elimination of wrinkles. In unexposed regions, wrinkles are also affected and rearranged perpendicular to the exposed area during the ordering.

**Figure 4:**
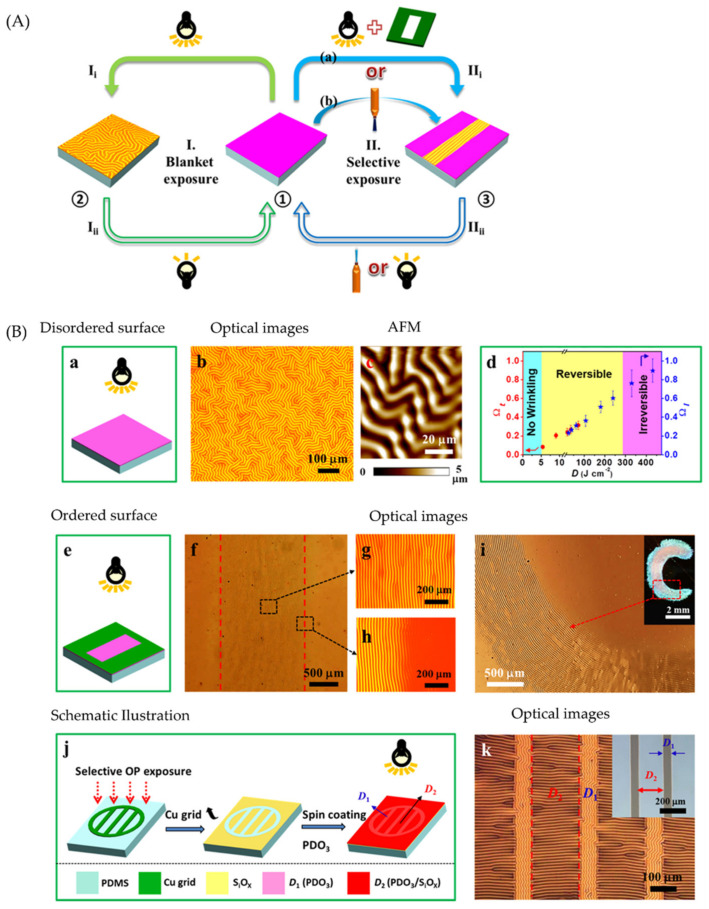
(**A**) Schematic illustration of the all-optical reversible surface patterning strategy and (**B**) Disordered/ordered surface wrinkling induced by the controlled exposure of the PDO3/PDMS bilayer to a high-intensity white light. Schematic illustration (a), optical (b), and atomic force microscopy (AFM) height (c) images of labyrinth wrinkles induced by blanket exposure. (d) The dependence of the aspect ratio (Ω_t_ and Ω_I_) of labyrinth wrinkles on the exposure dose D from different exposure times t and light intensity I employed, respectively. Schematic illustration (e) and optical images (f–i) of oriented wrinkles resulting from selective exposure through an oblong-shaped mask with the width of 1.5 mm and the length of 6 mm (f–h) or “C”-shaped mask with the hole width of 1 mm (i). Schematic illustration (j) and optical image (k) of dually oriented wrinkles induced by blanket exposure, where the substrate was pre-exposed to oxygen plasma through a copper grid (insert of (k)). Reproduced with permission from reference [43]. Copyright 2019, American Chemical Society (ACS).

Cao, Fery, and Lu et al. [43] showed a methodology to trigger various wrinkling patterns on the surface of azo-polymer composite substrate systems capable of switching between flat and wrinkled states via light incidence. The reversible patterning strategy is illustrated in Figure 4a, which is divided into two alternative routes. Route I is based on the exposure of a planar PDO_3_/PDMS bilayer system to a high-intensity white light (˃0.4 Wcm^−2^), resulting in randomly distributed wrinkles. Notably, the surface wrinkles are gradually erased using a low light intensity of 15 mW cm^−2^, allowing the recovery of the initial flat surface. In route II, a selective exposure of high-intensity light is applied by utilizing a mask or a high-intensity 450 nm laser beam with controllable parameters such as speed and movement. Figure 4b shows a schematic illustration of the approaches followed to fabricate dually oriented hierarchical wrinkle patterns. The soft/rigid boundary was generated by the PDMS substrate’s selective oxygen plasma treatment (OPT) through copper grids, followed by spin-coating the PDO_3_ film. As expected, hierarchically ordered wrinkles are formed after blanket exposure with parallel and perpendicular alignment in the softer D1 and harder D2 boundaries.

**Figure 5:**
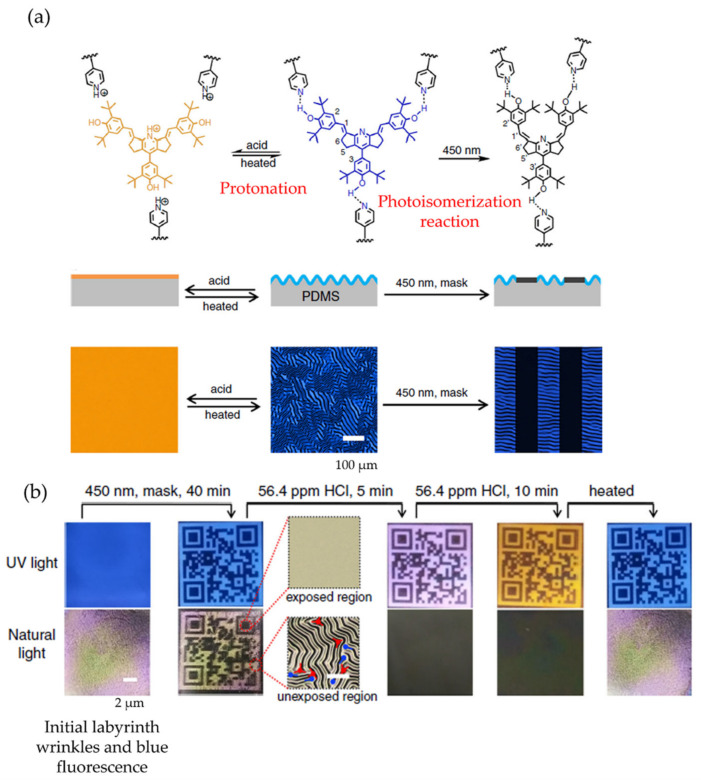
(**a**) Strategy to produce dynamic wrinkled patterns with tunable fluorescence using P4VP-nBA-S/DSP-OH, characterized by laser scanning confocal microscopy (LSCM), and (**b**) photographs of QR code based on wrinkled and fluorescent patterns for anti-counterfeiting applications. Reproduced with permission from reference [44]. Copyright 2020, Springer Nature.

Another exciting example of reversible stimuli-responsive wrinkled patterns triggered by visible light was reported by Ma and Jiang et al. [44], who reported a reversible dual pattern that responded to light and acid treatment. The material employed is a supramolecular network comprising a copolymer containing pyridine (P4VP-nBA-S) and hydroxyl distyrylpyridine (DSP-OH), which exhibited blue fluorescence on its surface. It is noted that the wrinkle morphology and fluorescence color can be regulated by the isomerization of DSP-OH triggered by visible light or acid treatment (Figure 5a). Experimentally, the polymer blend was spin-coated onto a PDMS substrate, producing a stiff laminate with a thickness of 100 nm at the top. Thermal treatment at 110 °C introduced compressive stress into the system generating the wrinkle surface structure. The resulting hierarchical patterned surface becomes smooth and wrinkled reversible on irradiation with 450 nm visible light or acid treatment (hydrogen chloride gas). Simultaneously, its fluorescence changes dynamically from blue to orange-red due to the chemical structure’s strong solid push-pull electronic effect. Figure 5b shows a possible application as an anti-counterfeiting platform related to a simple thermal treatment. After irradiation with 450 nm light through a QR code-shaped photomask, the wrinkled pattern of the QR code with blue fluorescence was carried out. When the sample was exposed to 56.4 ppm HCl vapor for 5 min, the wrinkles flattened, and the QR code fluorescence changed to purple or orange (10 min). After heating to evaporate the HCl, the wrinkles reappeared, and the QR code fluorescence returned to blue.

At the same time, Zhang, Jiang, and Lu, et al. [45] have described a simple and effective strategy to control the transformation of the wrinkle structure and the resulting hierarchical micropatterns using an azo-type poly(disperse orange 3) (PDO3) film as a rigid top layer with different rigid interlayers (polystyrene and silica-type SiOx) bonded to a PDMS substrate.

A rigid photoinert interlayer induces a tension-relaxation effect in photomodulated wrinkles. This phenomenon is highly dependent on the material properties of the photoinert interlayer. Consequently, the wrinkles were transformed into highly oriented patterns in unexposed areas after selective exposure. On the other hand, they were either wholly erased or evolved into wrinkles of shorter wavelengths in the exposed part. These studies offer alternative routes for preparing hierarchical surface patterns in multilayer functional devices.

**Figure 6:**
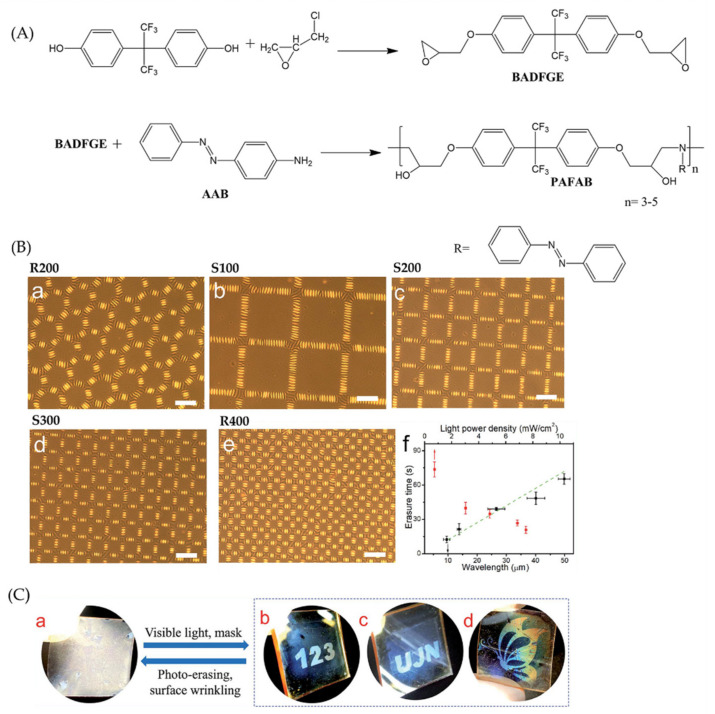
(**A**) Schematic illustration of the process used to synthesize the epoxy-based oligomer (PAFAB), (**B**) optical images of the patterns of wrinkles on the PDMS/PAFAB bilayer system irradiated with visible light through different copper grids (R200 (a), S100 (b), S200 (c), S300 (d), and R400 (e)) and their dependence of the optical erasure time on the light power density and the wrinkle wavelength (f) and (**C**) applications of the PDMS/PAFAB wrinkling system for rewritable information storage as a result of selective exposure to visible light: as-fabricated wrinkle-forming surface (a); various patterns of wrinkles obtained by selective exposure via different photomasks (b–d). Reproduced with permission from reference [46]. Copyright 2020, Royal Society of Chemistry (RSC).

Similarly, Zhang and Zong et al. [46] have published a simple visible light illumination method to fabricate a wrinkle-forming innovative surface with photo-controllable hierarchical surface patterns using an epoxy-based oligomer possessing azobenzene. The epoxy oligomer was synthesized by ring-opening polymerization of bisphenol AF diglycidyl ether (BADFGE) with p-aminoazobenzene (AAB). The elicitation route is shown in Figure 6a. The appearance of surface wrinkling occurred through the circulation of heating/cooling cycles and photo-conformation due to photo softening and the release of stress induced by the photoisomerization of azobenzene. When illuminated with visible light by selective exposure of selected areas, wrinkles could be rapidly photo-eliminated to give rise to hierarchical surface patterns.

Furthermore, the time required to erase the exposed wrinkled regions depended highly on the wrinkles’ wavelength and the light’s power density (Figure 6b). Finally, as presented in Figure 6c, several patterns could be visualized by employing selective exposure to visible light. Unexposed regions that formed wrinkles showed lower transmittance and intense scattering.

NIR light could also trigger reversible transformations of stimuli-responsive wrinkled patterns. For example, Jiang et al. [47] presented a simple and general strategy for forming NIR-responsive dynamic wrinkle patterns with excellent reversibility and responsive capacity. PDMS elastomers containing a small amount of CNT (CNT-PDMS) served as the substrate for the bilayer system with various functional polymers as rigid top layers. NIR irradiation can again induce thermal expansion of the CNT-PDMS substrate, resulting in a decrease in the compressive stress of the bilayer system and the elimination of wrinkles. After removing the NIR source and cooling it to room temperature, the smooth surface returns to the original wrinkles. This research demonstrates that this strategy can be applied in situ to tune surface properties on demand. The same group [48] reported another method to elaborate a reversible double-dynamic pattern based on a bilayer system that allows simultaneous control of wrinkle topography and fluorescence. A copolymer containing anthracene (AN) and naphthalene diimide (NDI) (PAN-DNI-BA) was used as a rigid top layer. The emerging patterns assume reversible changes between the smooth and wrinkled states upon irradiation with light and subsequent heat treatment.

The fluorescence reversibly changes from red to white and finally to blue-green because of the tunable charge transfer interaction between AN and NDI in the PAN-DNI-BA copolymer. The smart surfaces with dynamic hierarchical wrinkles and fluorescence were achieved by selective irradiation with photomasks. They can be employed for potential applications in smart displays and anti-counterfeiting purposes. Similarly, Jiang, Russell, and Zhang et al. [49] present a promising method for producing long-range ordered patterns by exposing a photomask on a thin polymer film. A thin film of a light-sensitive polymer containing anthracene (LAP), supported on a polyethylene terephthalate (PET) film or glass sheet, was exposed to 365 nm UV light using a photomask for a specific time to initiate photodimerization of the anthracene. Thus, LAP photodimerizes to DLAP, and a gradient in DLAP concentration is generated, where the top layer of the film is highly crosslinked and has a higher elastic modulus than the sub-surface regions. Pattern control was carried out using a photomask, resulting in a compressive force only in areas exposed to UV light. This process gives rise to wrinkling or buckling while the unexposed area remains flat. Similar studies were reported by Jian, Li, and Lu et al. [50], incorporating an amorphous azobenzene-containing PDO_3_ film as an intermediate layer to design a photosensitive multilayer-based film/substrate system.

Jiang et al. [51] reported a simple but effective anti-counterfeiting strategy using NIR-driven dynamic wrinkle pattern memory surfaces that can be used as dynamic biomimetic fingerprints. The generated wrinkle pattern works similarly to a fingerprint, which allows a higher level of anti-counterfeiting to be implemented, thanks to the non-deterministic process, unpredictability, and randomness of the wrinkle formation process. These dynamic wrinkles were fabricated using a bilayer system with CNT-PDMS (small amount of carbon nanotubes-CNTs- in polydimethylsiloxane) as an elastic substrate and a photo cross-linkable copolymer containing anthracene (PAN) as a rigid top layer, which was rotationally coated onto the elastomer. This dynamic fingerprint-like wrinkle’s key point is that the wrinkle’s minutiae remain unchanged during multiple cycles of wrinkle erasure/regeneration. When using heat treatment directly or IR irradiation, the imbalance between the bilayer’s moduli and thermal expansion ratios produces compressive stress, resulting in randomly distributed wrinkled patterns, which possess fingerprint-like minutiae: ridge termination and bifurcation [52]. These stress-controlled wrinkles are reversible and sensitive to the bilayer’s thermal expansion caused by IR irradiation.

**Figure 7:**
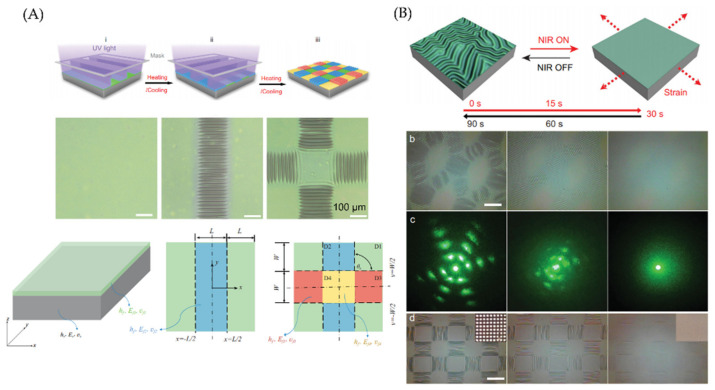
(**A**) Schematic representation of the light-controlled wrinkled pattern fabrication process via a lithographic approach, using 365 nm sequential UV from a 200 μm strip array photomask. (**B**) Illustration of the NIR-driven ordered wrinkles serving as a dynamic light grafting. (b) Optical images of the wrinkle disappearance/regeneration process by the NIR on/off switch. (c) The corresponding light diffraction patterns. (d) Optical images of Au-functionalized wrinkle disappearance/regeneration process by NIR on/off switch. Reproduced with permission from reference [53]. Copyright 2020, Science Publishing & Media Ltd. (Science Press).

In addition, they developed a fluorescent label that presents dynamic wrinkles for anti-counterfeiting some difficult-to-recognize objects (ancient Chinese Ruyao porcelain from the Song dynasty is tricky because the porcelain surface is usually smooth). The study was carried out by replacing PAN with anthracene (AN) and naphthalene diimide (NDI) motifs with a copolymer (PAN-NDI) as a top layer. The evolution of wrinkles was recorded by surface analysis using fluorescence microscopy. The same group [53] presents a methodology to regulate the wrinkle topographies by precisely controlling the spatial distributions of the modulus and boundary conditions of the top layer in bilayer systems composed of an anthracene-containing copolymer (PAN-BA) on a PDMS elastomeric substrate. The authors state that by this method, they could fabricate and regulate the wrinkles in 2D (Figure 7a).

By irradiating with 365 nm ultraviolet light for 15 min by a photomask strip, heating it at 70 °C for 30 min, and cooling, 1D wrinkles were spontaneously generated in the exposed domain. Posteriorly, the photomask was rotated horizontally with a certain angle. Thus, the 1D ordered wrinkled surface (one direction on a two-dimensional surface) was again exposed for 15 min, allowing 2D wrinkle formation (multi-direction on a two-dimensional surface) through heating at 70 °C and cooling treatment at 20 °C. This exposure sequence form two strip-like domains (two directions of Poisson’s effect in the double exposure domain D4). Parallel, NIR was utilized to tune the resulting wrinkle by controlling the bilayer system’s compressive strain; mainly, PDMS-containing carbon nanotube (CNT-PDMS, 0.05 wt%) was used as a substrate for the bilayer system. The formed wrinkled pattern can be erased in situ and in real-time due to the high efficiency of photon-to-thermal energy conversion (Figure 7b). Their thermal expansion can be reversibly controlled by applying 808 nm NIR irradiation to the CNT-PDMS substrate. When the wrinkled surface was irradiated with NIR, the diffraction patterns of 3rd generation disappeared rapidly within 30 s. By removing the NIR radiation, these diffraction patterns were gradually restored to their original state due to the regeneration of the wrinkles.

**Figure 8:**
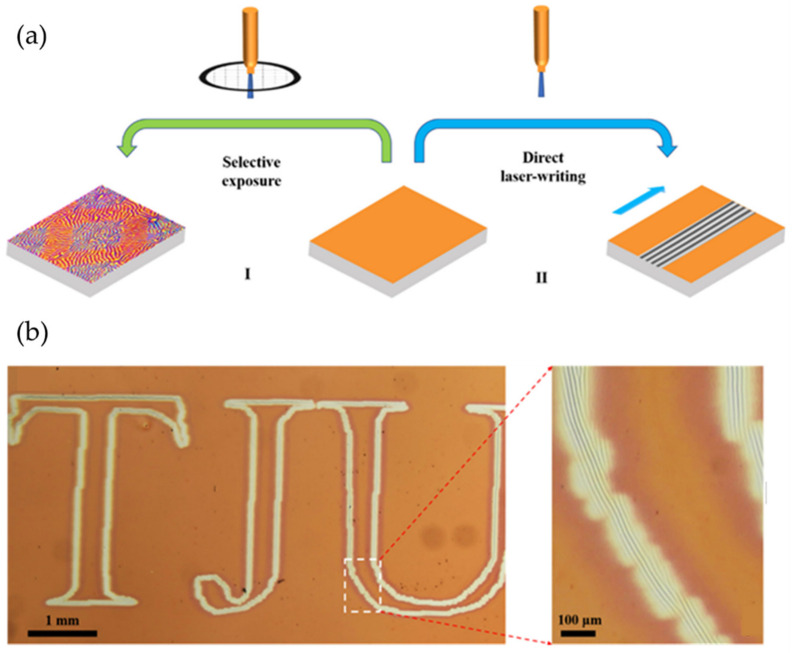
(**a**) Representative schematic of the wrinkle-inducing pathways upon exposure of a (PDO 3-PS)/PDMS bilayer to a 450 nm laser with a photomask (I) or a moving laser (II) and (**b**) Optical image of trajectory-guided oriented wrinkling (TJU) of (2% PDO3 - 2% PS)/PDMS induced by a moving high-intensity laser beam (e.g., 16.20 W-cm^−2^/1.0 mm-s^−1^). Reproduced with permission from reference [54]. Copyright 2022, MDPI.

On the other hand, Bowen Wang et al. [54] reported a simple strategy to create micro/nanopatterns on a surface composed of a film/substrate bilayer based on a non-photosensitive azo-polymer and a soft substrate. In this system, light exposure generated wrinkled patterns on the material’s surface. This strategy is illustrated in Figure 8a, which entails two alternative paths. In the first alternative, the planar (POD3-PS)/PDMS bilayer is exposed to a 450 nm laser beam selectively using the copper grating as a photomask, generating labyrinth wrinkles. The second method is based on a dynamic surface exposure that directs the pattern orientation perpendicular to the motion of the 450 nm laser. Therefore, the unexposed surface areas will maintain their basal state. As a result, micropatterns can be formed by writing the surface with the laser, in which the trajectory of this light visualized in Figure 8b is controlled, observing these parallel patterns in the drawn letters.

**Figure 9:**
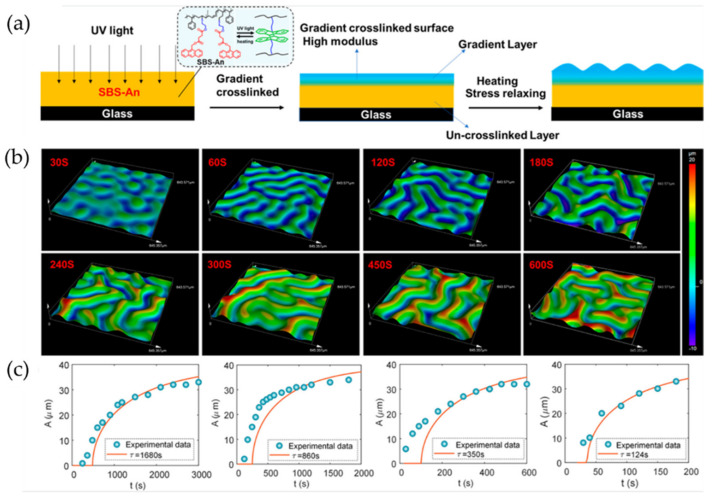
(**a**) Schematic representation of the self-wrinkling process, (**b**) Evolution of wrinkle morphology at 120 °C for different times, and (**c**) Morphology evolution at different temperatures (100, 110, 120, and 130 °C). Reproduced with permission from reference [55]. Copyright 2022, American Chemical Society (ACS).

Bai et al. reported the design of a viscoelastic monolayer composed of photosensitive poly(styrene-block-butadiene-block-styreneSBS-An) [55]. The viscoelastic role of the polymer layer is fundamental to understanding the wrinkle growth mechanism. The substrate does not have enough capacity to store energy, which is why the wrinkle formation process, being a kinetic process, is time-dependent. Figure 9a illustrates the wrinkle formation process, in which SBS-An was coated onto a rigid substrate and irradiated with 365 nm UV light. As a result, a crosslinking gradient was formed on the film surface. Figure 9b shows the evolution of the wrinkled morphology when the material is exposed to high temperatures (120 °C). First, a smooth surface is obtained; at 30 s, the first spherical cup patterns are observed, rapidly growing into laberinth-like patterns. These patterns increase amplitude and wavelength with time (from 60 to 600 s). Figure 9c shows the evolution of wrinkles with different temperatures (100, 110, 120, and 130 °C), showing an increase in amplitude with treatment time.

**Figure 10:**
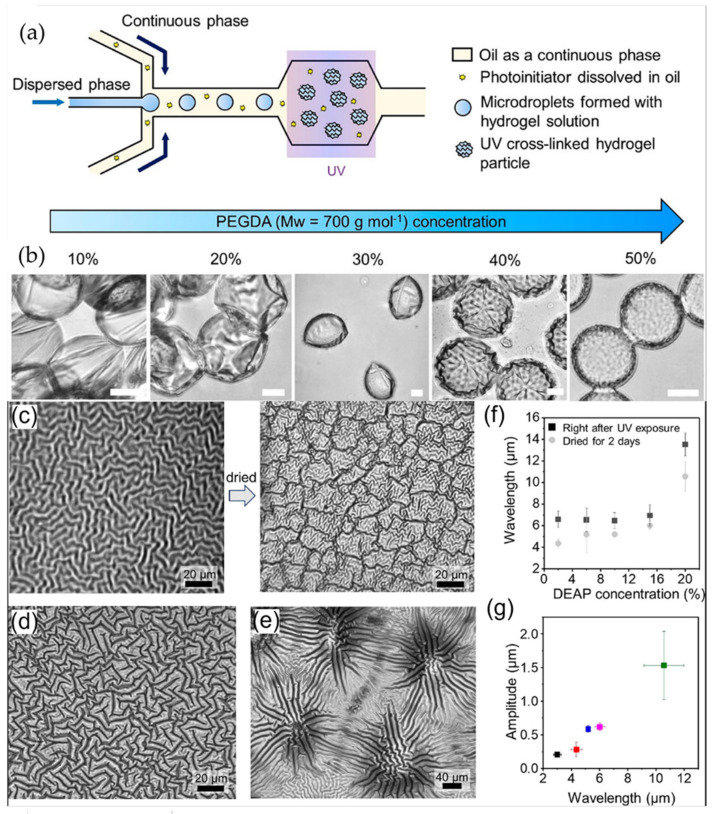
(**a**) Schematic representation of wrinkled hydrogel particles formation, using an oil-water interface, (**b**) Effect of polymer concentration on hydrogel morphology, (**c**–**e**) Labyrinth wrinkle formation and its contraction, and (**f**,**g**) Effect of photoinitiator in wrinkles dimensions. Reproduced with permission from reference [56]. Copyright 2021, American Chemical Society (ACS).

Um et al. reported a one-step strategy to induce wrinkle formation in hydrogels using oil-water interfaces. The preparation method of wrinkled particles is illustrated in Figure 10a, where an oil phase containing the photoinitiator 2,2-diethoxyacetophenone (DEAP) flowed through microchannels. Subsequently, the oil phase flows to an aqueous phase containing polyethylenglycol diacrylate (PEGDA_700_) polymer, forming droplets that travel to a reservoir to be exposed to UV light, producing crosslinked hydrogels. The morphology of the photoreticulated hydrogel particles will vary depending on the volumetric concentration of PEGDA_700_ in the aqueous solution (Figure 10b). Solution concentrations lower than 20% *v*/*v* tend to form thin capsules with wrinkles at the surface, while those containing 30% *v*/*v* were similar to coffee beans with folds. Spherical particles without liquid content and prominent surface wrinkles are observed for concentrations higher than 30%.

Moreover, the authors observed that labyrinth wrinkles were produced for concentrations of DEAP at 2% (Figure 10c). After washing and drying, either small folds or ridges were created on the hydrogel surface. For concentrations of 6% to 10%, the labyrinth shape of the patterns was preserved (Figure 10d). However, for concentrations of 20%, no labyrinth patterns are obtained, but rather flower-like patterns are depicted (Figure 10e). The effect of DEAP concentration on the wrinkle wavelength (Figure 10f) and amplitude (Figure 10g) was also determined.

In the following table (Table 1), most of the examples for light-responsive materials previously mentioned are summarized, emphasizing the base material, methodology used to fabricate the wrinkled patterns and the possible application of the device. The examples were ordered according to the type of light used in each case (visible, UV, or NIR light). As is possible to observe, in general, independently of the material used, the process used to fabricate the wrinkles is similar in all the cases.

### 2.2. Alternatives for the Fabrication of pH-Responsive Wrinkled Surfaces

Several examples have been reported where the interface responds to stimuli like pH or humidity. Sun et al. [57] reported on creating an intelligent film that can reversibly change its wrinkling pattern in response to changes in ambient humidity. The films were formed by depositing hydrophobic SiO_2_ nanoparticles (NPs) on wrinkled polyelectrolyte surfaces. Experimentally, the substrate (glass and silicon wafers) was treated and bathed in an aqueous solution of poly(diallyl dimethyl ammonium chloride) (PDDA) to achieve a positively charged surface. Subsequently, the PDDA-modified substrate was immersed in an aqueous solution of poly(acrylic acid) (PAA) and poly(allylamine hydrochloride) (PAH), thus generating a multilayered film. On top of the films (PAA/PAH), a SiO_2_ NP layer was formed via immersion. This cycle was repeated to obtain multilayers of (SiO_2_/PAH)*m films (where m refers to the number of deposition cycles and a middle number means that SiO_2_ NPs are in the last layer). The multilayer was then exposed to thermal crosslinking and subsequent deposition of 1H,1H,2H,2H-perfluoro-octyltriethoxysilane (POTS) on (SiO_2_/PAH)*m (PAA/PAH)*30 films by a chemical vapor deposition (CVD) process.

**Figure 11:**
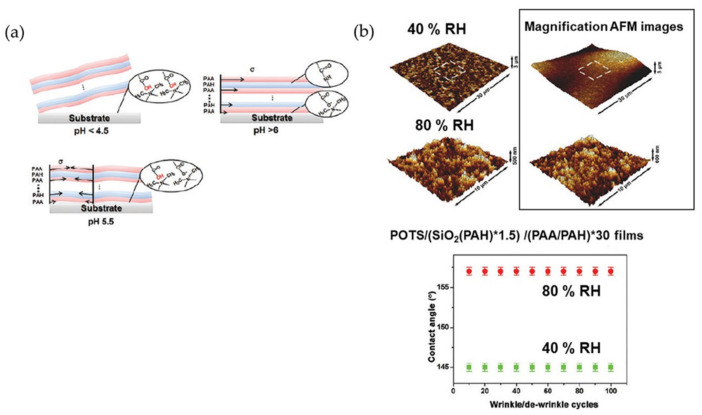
(**a**) Schematic illustration of the interaction of the PAA/PAH films with the PDDA-modified substrate after being immersed in water with different pHs (˂4.5; 6˃ and 5.5.) and (**b**) AFM images and water contact angle changes of the films during repeated de-wrinkle/wrinkle process in 40% RH and 80% RH. Reproduced with permission from reference [57]. Copyright 2016, Springer Nature.

Figure 11a shows wrinkled surface patterns formed based on the ionization ratio of carboxylate groups of the bottom PAA layer of the (PAA/PAH) films at different pHs. The PAA and substrate interaction seems to be reduced when the films are introduced into an acidic aqueous solution (pH ˂ 4.5). After water adsorption, the compressive stress of the film exceeds the confining stress of the substrate, leading to a complete exfoliation of the film from the substrate. Likewise, by modifying the solution to pH 6.5, the interaction between PAA- and PDDA-modified substrates cannot be weakened by immersing the films in a more basic aqueous solution. In this case, the compressive stress generated by the film’s swelling is lower than the confining stress of the substrate, which prevents the formation of wrinkles. When the crosslinked (PAA/PAH) films are in a water solution with 5.5 pH, the interaction of the films with the PDDA-modified substrate is moderately weakened. These interaction threshold values allow for partial separation of the films from the underlying substrate upon water adsorption. The separated areas of the film tend to crease, while the areas on the left adhere to the underlying substrates. Thus, wrinkles form in the crosslinked films (PAA/PAH). Figure 11b shows the creation and disappearance of the wrinkled surfaces as the films are subjected to different ambient relative humidities (80% RH and 40% RH).

In wrinkled films, structure colors appear due to the interference of reflected light on the wrinkled film surface. The films gradually form wrinkles, and the water contact angle increases with ambient humidity. Nanosized aggregates of SiO_2_ NPs are observed on the surface of the non-structured film, and the roughness is monitored by the AFM images of the wrinkles films increase to 256 nm. The researchers indicated that the surface roughness of wrinkled and unwrinkled films significantly influences their wettability and, therefore, can influence their degree of transparency. As a result, the composite can spontaneously change from transparent hydrophobicity to translucent superhydrophobicity through moisture-induced wrinkling/unwrinkling [57].

Similarly, Lu et al. [58] fabricated a film/substrate composed of (PAA/PEG)_n_/PDMS to investigate the time evolution of swelling/deswelling-induced wrinkle pattern formation. Poly(acrylic acid)(PAA) and poly(ethylene glycol) (PEG) were deposited on PMDS via a layer-by-layer dipping technique, followed by heating-induced covalent crosslinking. Later, two types of systems were fabricated, Pt/(PAA/PEG)_n_/PDMS and polystyrene (PS)/(PAA/PEG)_n_/PDMS. In situ optical microscopy imaging monitored the morphological transformation in the covalently bonded (PAA/PEG)n/PDMS system. Thus, when the wrinkled (PAA/PEG)n/PDMS system was immersed in an acidic aqueous solution of pH = 2.5, surface wrinkling patterns occurred, including forming primary wrinkles with a labyrinth-like structure and second-generation wrinkles, which exhibit ordered dimples of hexagonal arrangement and bridging maze patterns. The morphological evolution can be finely tuned considering the thickness of the film (PAA/PEG)_n_, the additional inert outer layer (Pt and PS), and the swelling directed by the solution pH. The introduction of an extra Pt layer, resistant to swelling/deswelling, improved the wrinkles’ stability, including the heat-induced primary and swelling-induced secondary patterns, leading to increased dimple periodicity. A similar evolution was obtained concerning the PS/(PAA/PEG)n/PDMS system during water swelling. Additional layer deposition was quickly applied to fabricate the desired multifunctional film/substrate systems for tunable stress-relief patterns and external applications.

**Figure 12:**
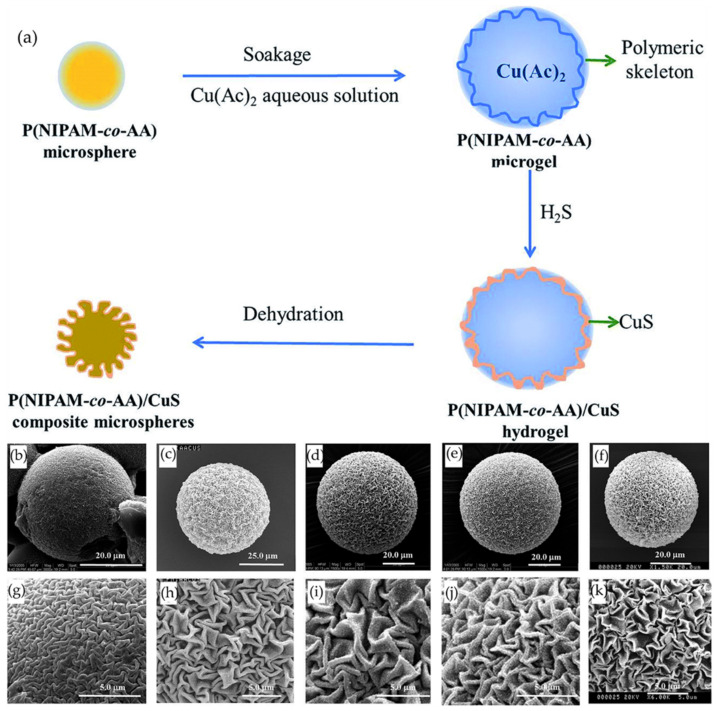
(**a**) Preparation of (P(NIPAM-*co*-AA)/CuS) microspheres, SEM micrographs P(NIPAM-*co*-AA)/CuS by adding HAc (pH = 3) (**b**,**g**), H_3_BO_3_ (**c**,**h**), ethylenediamine (**d**,**i**), pH = 5.6 (**e**,**j**) and pH = 9.2 buffer solution (**f**,**k**). Reproduced with permission from reference [59]. Copyright 2021, Royal Society of Chemistry (RSC).

Another interesting example was reported by Yang et al. [59], who synthesized poly(N-isopropylacrylamide-co-acrylic acid)/copper sulfide (P(NIPAM-co-AA)/CuS) composite microspheres which presented wrinkled zigzag surfaces formed through the in situ biomimetic mineralization reaction between H_2_S and Cu^+2^ (Figure 12a). The main objective is to generate wrinkled patterns on a hydrogel film by deposition of a sulfide on the surface, where the amount deposited and the distribution of the sulfide affect the surface morphology obtained by forming these patterns. The effect of acidity and ionic strength of the Cu(Ac)_2_ solution were analyzed using different solutions of Cu(Ac)_2_-HAc, Cu(Ac)_2_-H_3_BO_3_, Cu(Ac)_2_-ethylenediamine and buffer solutions of pH = 5.6 and pH = 9.2. In the case of microgels in Cu(Ac)_2_-HAc solution, small wrinkles are formed due to the degree of acidity of HAc (Figure 12b,g). In contrast, hydrogels in Cu(Ac)_2_-ethylenediamine solution (Figure 12d,i) and Cu(Ac)_2_-H_3_BO_3_ (Figure 12c,h) present a similar structuration between them. When these microgels are under acidic (Figure 12f,k) or basic (Figure 12e,j) buffer solution, they present smaller wrinkles because the buffer influence eliminates the effect of proton release during precipitation. However, the wrinkles in a basic buffer tend to increase their dimension compared to the acidic medium.

Table 2 summarizes the previously mentioned examples for pH-sensitive materials, their methodology for fabricating the wrinkled patterns, and their possible application. The examples were ordered depending on their responsive pH range used in each case. Unlike the examples in the previous section (Table 1), the process of obtaining the wrinkles widely differs in this case.

### 2.3. Wrinkle Formation in Systems Incorporating Temperature-Responsive Polymers

Another way to produce stimuli-responsive wrinkled patterns is by using polymers that display temperature-responsive properties. Zhang and Sun et al. [60] studied three types of film-substrate bilayer devices that consist of a stiff hydrophilic polyvinyl alcohol (PVA) film tightly adhered to a hydrophobic soft PDMS substrate (PVA-PDMS film-substrate systems). Some samples exhibit reversible wrinkles forming-disappearing behavior, while others only temporarily show wrinkles formation at the beginning of the moisture exposure, which disappears during the subsequent moisturizing and never reappears in the following drying/remoisturizing. Interestingly, another sample could be prepared that evidenced the formation of permanent wrinkles once exposed to moisture, regardless of the following moisturizing and drying conditions. The design was performed by adjusting precisely the PVA thin film’s moisture-dependent responsive properties by controlling the thickness and crosslink gradient and the film-to-substrate thickness ratio. The evolution of wrinkle formation was manifested as the change in the transparency of the bilayer material.

Similarly, Tokudome and Takahashi et al. [61] report the fabrication of adaptive microarchitecture that exhibits a particular peristaltic motion by employing hydrogel actuation on bilayered materials. They designed and fabricated a bilayer structure of poly(N-iso-propylacrylaimide) PNIPAM and hybrid silica. The thickness of the hybrid silica layer was tuned from 680 nm to 1.5 μm using Pluronic F127 as a viscosity improver. 3-(methacryloxy)propyl triethoxysilane was used as a silica source to allow copolymerization with NIPAM and a crosslinker (N,N′-methylenebis (acrylamide), MBA) at the interface of the heterogeneous layers. PNIPAM undergoes shape changes at the lower critical solution temperature (LCST) [62], and therefore, submillimeter wrinkles appear and disappear as a function of the changing environmental temperature. The wrinkles were found to show a peristaltic motion on cooling from 36 °C to room temperature. The periodic length of wrinkles (λ) increases with the crust layer’s thickness (t). It also shows that the peristaltic motion was achieved on a patterned hybrid silica layer (triangle-shaped patterning) placed on top of the PNIPAM. Oriented anisotropic wrinkles form on cooling because the patterning can accumulate anisotropic stress. Stress relaxation occurs in the *x*-direction, and the stress is uniaxially accumulated in the *y*-direction to form anisotropic parallel wrinkles.

**Figure 13:**
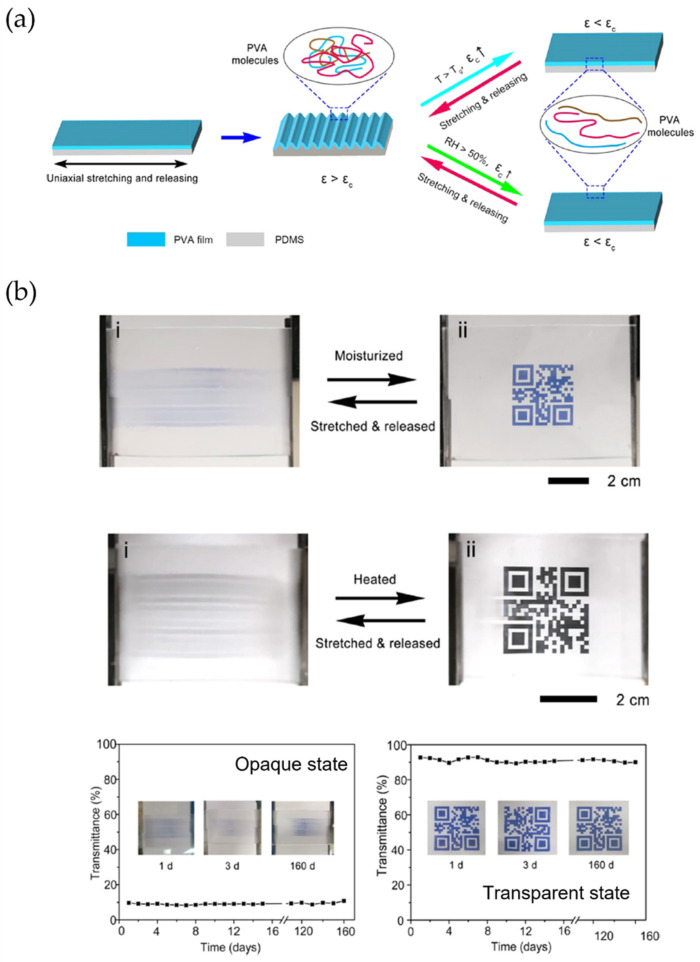
(**a**) Schematic illustration showing the fabrication process and reversible wrinkling/dewrinkling mechanism of moisture and temperature dual-responsive surface wrinkles on the PVDA/PMDS film. (**b**) Dual stimuli-sensitive smart windows. Digital photos of humidity-driven smart windows (top, i: stretched and released, ii: moisturized). Digital photos of temperature-driven smart windows (middle i: stretched and released, ii: heated) and transmittance of smart windows as a function of storage times (bottom). Reproduced with permission from reference [64]. Copyright 2019, American Chemical Society (ACS).

Xiao and Wang [63] studied heat-responsive shape memory polymers (SMP) bonded to PDMS substrates to form bilayer systems able to form surface microstructures. Similar examples were reported by Wang et al. [64], who showed a new strategy for developing moisture and temperature dual-responsible surface wrinkles on PVA/PDMS bilayer films by rational design of the PVA skin layer. The surface wrinkles are produced by uniaxially stretching and subsequently releasing the bilayer films because of the mismatching in elastic moduli of the PVA and PDMS and return to the flattened state when exposed to moisture or high temperature (Figure 13a). For the formation of the initial surface wrinkles, the PVA/PDMS films were maintained under the 70% RH conditions for 10 min before stretching, applying then 15% strain (ε) for 10 s and finally releasing the strain. To decrease the modulus of PVA by moisture or temperature, increase ε_c_ for the bilayer film, resulting in ε ˂ ε_c_ and the disappearance of surface wrinkles (Figure 13a).

Figure 13b shows digital photos of the smart windows actuated by moisture and temperature, respectively. The wrinkling films are highly opaque, which can hide the films’ objects; when the bilayer films were exposed to moisture at 80% RH or temperatures over 70 °C, the films rapidly changed to a transparent state to show the object behind clearly. Finally, it shows the wrinkles/dewrinkling film actuated by moisture and temperature stability with high transmittance for over five months at ambient room conditions.

**Figure 14:**
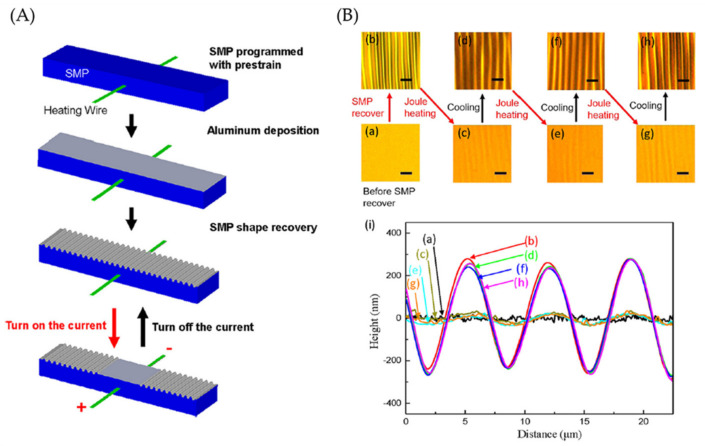
(**A**) Schematic illustration of the fabrication process to fabricate wrinkled patterns on SMP and locally flatten the surface using joule heating and (**B**) optical microscopy images of the surface in the reversible and repeatable tuning process (a)–(h) and the corresponding profiles (i) for Joule heating with a current of I = 0.45 A and heating for 3 min and cooling for 5 min. Reproduced with permission from reference [65]. Copyright 2019, AIP Publishing LLC.

Another example was developed by Xiao et al. [65], who utilized self-assembled surface wrinkling induced by SMPs and localized thermal expansion caused by Joule heating. SMP was synthesized by mixing tert-butyl acrylate (tBA) and crosslinker poly(ethylene glycol) dimethacrylate (PEGDMA) at a ratio of 4:1 and 2,2-dimethoxy-2-phenylacetophenone as a photoinitiator. The solution was injected into a mold with a piece of heating fire (FeCrAl alloy) placed at the center of the mold and cured under a UV lamp. Figure 14a shows a schematic representation of the process followed to obtain temperature-responsive wrinkled patterns; firstly, the SMP substrate was uniaxially pre-stretched with 5% strain (60 °C), using a dynamical mechanical analysis tester. After the sample was quenched to 25 °C, the strains were released, and the temporary and programmed shape was retained. Then, a layer of thin aluminum film of 100 nm was deposited on the programmed SMP substrate’s surface using a thermal evaporator. Afterward, the SMP was heated to 60 °C to induce shape recovery in the substrate, leading to wrinkling aluminum film. This behavior can be observed in Figure 14b through optical microscopy images of the surface morphology during the reversible tuning process. According to the profile, the smooth and wrinkled states match well with their correspondents in different cycles, demonstrating excellent repeatability and reversibility.

**Figure 15:**
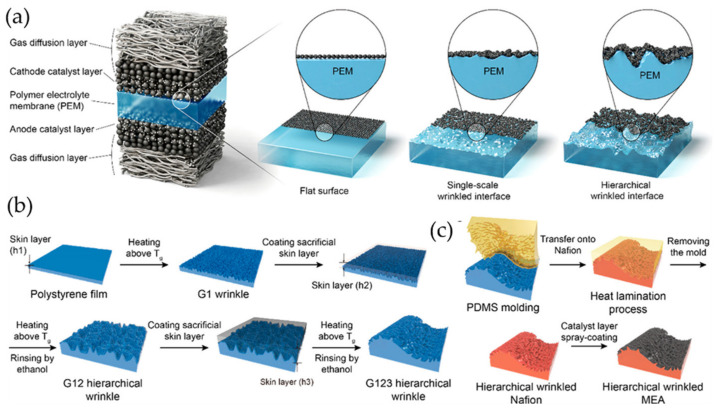
Schematic illustrations of hierarchical wrinkled PEM/CL fabrication. (**a**) The types of hierarchical wrinkled PEM/CL interfaces in PEMFC. (**b**) Fabrication of hierarchical wrinkles through repetitive heat-induced wrinkling processes and (**c**) Transfer of wrinkled hierarchical structures to Nafion membrane by thermal lamination and subsequent CL coating. Reproduced permission from with reference [66]. Copyright 2022, American Chemical Society (ACS).

Lee et al. [66] presented the fabrication of an interface composed of a polymer electrolyte membrane (PEM) and a hierarchically wrinkled catalyst (CL) layer (PEM/CL) in a PEM fuel cell (PEMFC) by bottom-up methods (Figure 15). Heat-induced repetitive mechanical deformations between a rigid top skin and a soft bottom substrate were used to fabricate these wrinkles. In Figure 15a, three different types of wrinkled PEMs were prepared. Heat-induced mechanical deformations were used to obtain these, with the thermal expansion mismatch between the bilayer causing deformation in the whole area by stress release. In Figure 15b, hierarchical nested wrinkled patterns (1st generation, which they named “G1”, similarly 2nd generation “G2” and 3rd generation wrinkle patterns “G3”) are fabricated using an argon plasma-treated polystyrene (PS) film that is subsequently heated to a temperature above its glass transition (Tg) and allowed cool at room temperature. Figure 15c shows the process of transferring wrinkled patterns to surfaces. PDMS was used to obtain the inverse morphology of the hierarchical patterns of PS via mask peeling. To perform the Nafion thermal lamination process, the hierarchical PDMS was placed on this layer by applying heat and pressure to the membrane to print the PDMS wrinkles, obtaining a Nafion membrane with a hierarchical nested wrinkled pattern. Finally, this film was coated with a CL layer using spray coating to form the wrinkled PEM/CL interface.

In Table 3 , it is possible to observe a summary of the examples mentioned for thermo-sensitive materials used to create smart wrinkled patterns. The table also depicts the methodology used to fabricate the wrinkles and its possible application. In this case, and similarly to the one observed in Table 1, independently of the type of material used, the process employed to fabricate thermo-sensitive wrinkled patterns is quite similar.

### 2.4. Electro-Magnetical Response

Another way to create reversible sensitive wrinkled patterns is by using an electric field stimulus to induce changes in the material surface. The use of electrical sources is attractive due to its ability to be reversible, to have the potential to be miniaturized, to generate an immediate response, to have the scope to integrate functions such as auto-sensing devices, and the possibility of being able to control it in a programmable way. It has been displayed that Van der Waals forces and electrostatic interactions acting through an air gap (<100 nm) between a soft coating and a rigid plate can induce out-of-plane wrinkle instabilities, thus stabilizing stable patterns on top of the material.

Indeed, Van den Ende and Steeneken et al. [67] presented feasibility studies to trigger out-of-plane wrinkle instability by applying an electric field to an elastomer. The acrylate-based elastomeric layer is deposited, and UV cured on an indium tin oxide (ITO) coated glass panel. Thin gold top electrodes are deposited on top of the elastomer layers. When a high voltage is imposed between the upper and lower electrodes, a striking change is observed in the visible appearance of the sample. Thus, the destabilization of the wrinkles generates significant changes in the images reflected or transmitted through the elastomer-coated rigid plates.

**Figure 16:**
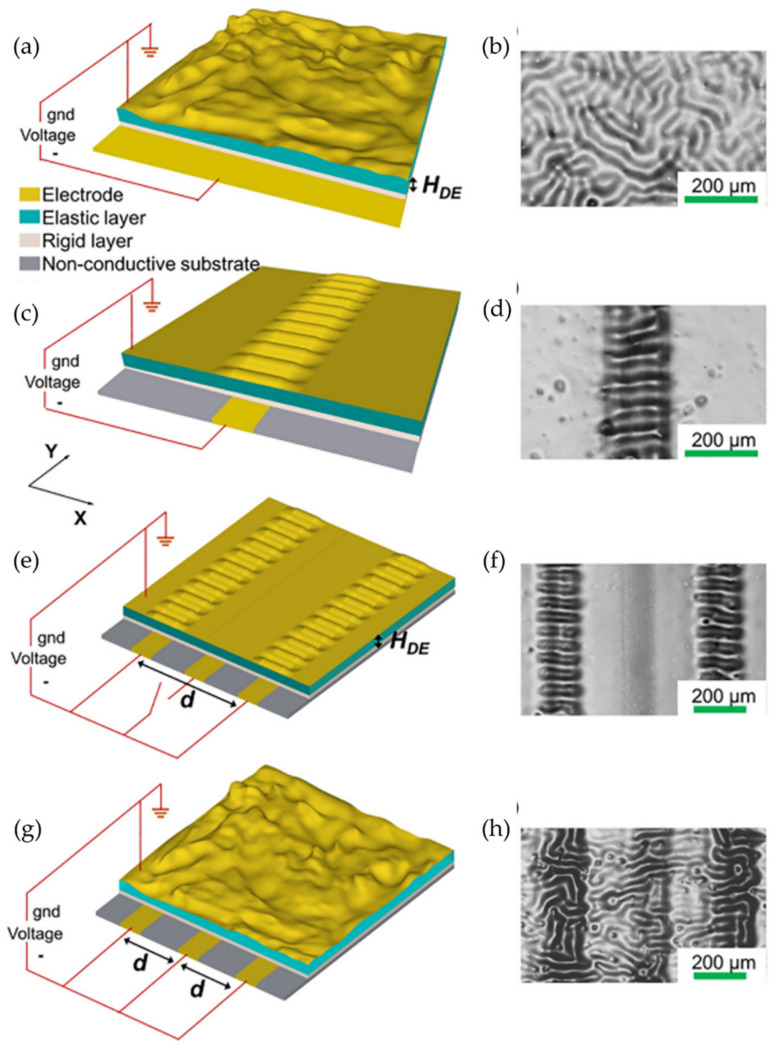
Schematics of the layered structure and operation with different electrode designs and the corresponding optical microscope images. (**a**,**b**) Randomly-oriented wrinkles, (**c**,**d**) When a single narrow electrode and (**e**,**f**) two electrodes spaced by a large distance *d* are used. (**g**,**h**) Randomly-oriented patterns when the spacing between narrow electrodes is smaller than a critical value. Reproduced with permission from reference [68]. Copyright 2020, Elsevier Ltd.

Similarly, Kar-Narayan and Smoukov et al. [68] reported the fabrication of controllable regular/irregular wrinkled patterns by a selective electron response system for a responsive polymer coating on underlying counter electrode patterns without requiring mechanical pre-stretch stimuli or the induction of anisotropy in the film. The electrode patterns were created by an aerosol jet printing method, introducing silver nanoparticles to form patterns on the bottom electrode. This research group used a rigid dielectric layer (Novolac bisphenol A epoxide, or SU-8) spin-coated onto the electrode layer (PDMS substrate). As a result, they determined that the polymer acts as a single electrode when there is minimal separation between the electrodes underneath it. The authors established that the regularity and direction of new patterns are influenced by the electrode spacing and defined parameters that regulate the alignment of the wrinkles. Figure 16 shows a schematic representation of fabricating wrinkled patterns with a selective electrical response. These results exhibited that applying this type of customizable wrinkle/electrode patterning with a light reflection-diffusion-grating device works. This technique produces complex responsive surfaces that possess a high electrical capacity.

On the other hand, Danas et al. [69] synthesized a polymer with a magnetic response (MRE, magnetorheological elastomer) which is attached to a passive substrate (soft non-magnetic polymer). Additionally, the MRE film includes iron particles isotropically and randomly embedded in the elastomer [70], resulting in no exhibition of magnetic hysteresis, producing that upon removing the magnetic fields, the wrinkled patterns tend to disappear. The analysis demonstrates that due to the robust magnetoelastic interaction, the high pre-compression, and the increasing magnetic field, the wrinkled pattern formed on top of the material tends to evolve from a smooth pattern to a complex one (commonly called crinkling). Thus, by increasing the pre-compression to λ_0_ = 0.8, it is possible to reduce the magnetomechanical wrinkling in the film. Further pre-compression increase generates a decupling between the mechanical and magnetical load effects. Danas et al. conclude that the friction in the film could interact with the magneto-mechanical wrinkling mode produce a decrease in the film thickness.

### 2.5. Mechanical (Stress/Stretch) Response

Mechanoresponse is an unusual phenomenon that occurs in some materials when a mechanical force is applied to them. This external mechanical stimulus presents several advantages, like manipulating the deformation time, direction, and strain/stress used. Recent studies have corroborated that mechanosensitive surface wrinkles possess dynamically adjustable, repeatable, and practical optical properties, which change their surface topography in response to mechanical stimulus. Indeed, Wang et al. [71] in 2018 reported a facile technique to fabricate PVA/PDMS bilayer films with various materials, including PVA, CS (chitosan), HEC (hydroxyethylcellulose), and the mixture of PVA and CS with mechanoresponsive surface wrinkles. The PVA/PMDS bilayer film surface wrinkled patterns exhibit a high optical response to minor mechanical stress, extensive optical switching cycles, and a wide range of transmittance modulation, attributing excellent mechanoresponsive optical switching properties. They test these systems as smart windows obtaining incredible results.

**Figure 17:**
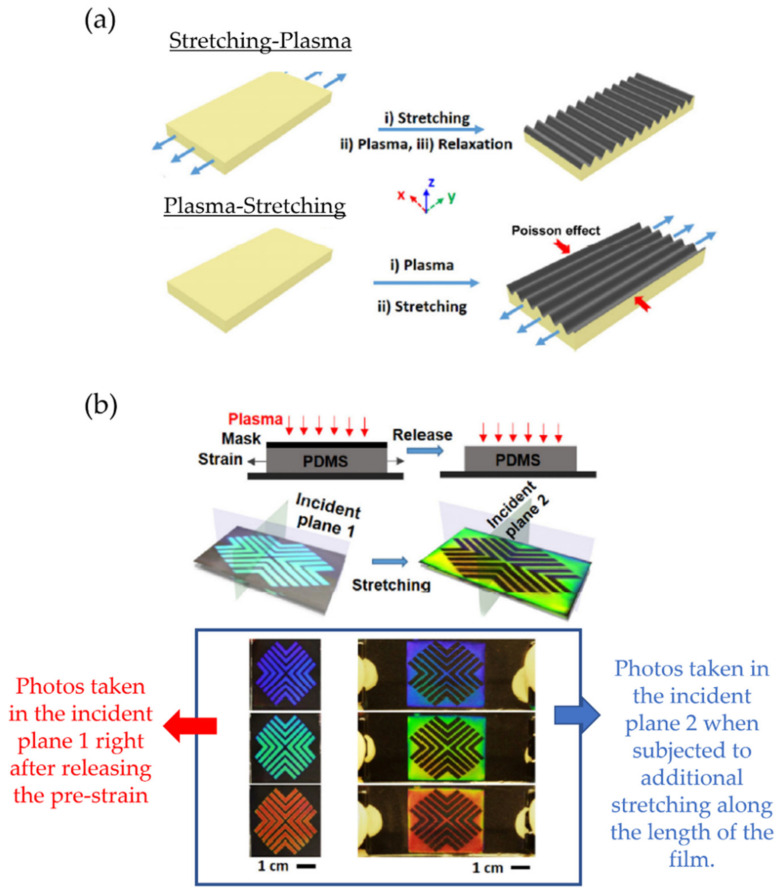
(**a**) Schematic illustration of the fabrication processes and (**b**) mechanochromic response and information encryption. Reproduced with permission from reference [72]. Copyright 2020, Springer Nature.

Some materials, such as mechano-chromic materials under the mechanical stimulus, exhibit a change in their optical properties, such as absorption, emission, and reflection; for example, Yin et al. [72] studied the wrinkling effect of PDMS films as a function of their thickness. In this work, the authors demonstrate that the periodicity and equality of wrinkles formed under mechanical stress are related to the thickness of PDMS-based films. Experimentally, films of different thicknesses were subjected to pre-stretching with a given tension and subsequently coating with a mask, followed by plasma treatment. Afterward, perpendicular wrinkles were obtained on the rigid surface (stretch plasma) after tension-release cycles. (Figure 17a). This new methodology makes it possible to obtain films with bright-colored finishes and predesigned colorimetric responses due to their mechano-chromatic response. Thus, the wrinkle formation mechanism was mapped in two regimes depending on the thickness threshold of around 1 mm. When these films had a thickness of less than 1 mm, wrinkled patterns with defects were formed, and their average periodicity was mitigated when the thickness approached the base value of 1 mm. On the contrary, when the thickness was larger than 1 mm, the wrinkles had a constant periodicity; this value did not vary with increasing thickness. The classical nonlinear finite mechanism can precisely predict it.

Figure 17b illustrates the stress and plasma treatment of PDMS films on the complementary and reversible coloring process. These treatments were performed by applying plasma with and without tension, including the mask. The remaining substrate was oxidized in the last process, obtaining a thin and stiff film. When these films underwent pre-coating, the patterns formed in the first treatment were erased. Meanwhile, uniform and aligned wrinkles were formed in the direction of the stretching during the second plasma treatment.

**Figure 18:**
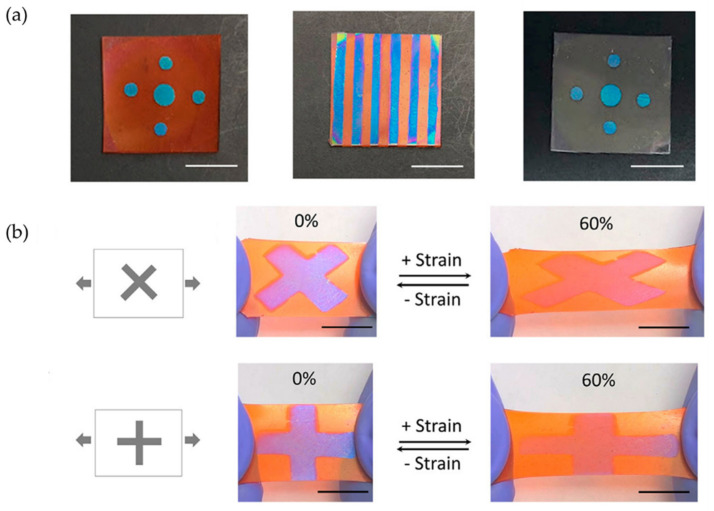
(**a**) Photographs of different color patterns generated in PVP-Ir-Dyed PDMS films with and without red dye and (**b**) stretching tests (from 0% to 60% strain) of the PVP-Ir-Dyed PDMS film, with load direction at 45° or 90° to the blue-colored cross pattern. Reproduced with permission from reference [73]. Copyright 2020, Elsevier Ltd.

Similarly, Oyefusi and Chen [73] developed an exciting method to fabricate reversible mechanochromic materials based on optical interference processes. They manufacture stretchable films with excellent low-cost mechanochromic properties through two complementary mechanical strategies based on shear and cracking (kirigami approach). This methodology obtained unique, anisotropic, and load direction-dependent mechano-chromatic properties by including kirigami. This new strategy allows the fabrication of polymeric systems with dynamic reversible mechanical properties with a high degree of sensitivity to small deformations (0–60%) without using external luminous stimuli, making them affordable and scalable. In this case, they select as mechano-chromic material a polyvinylpyrrolidone-iridium-polydimethylsiloxane (PVP-Ir-PDMS) trilayer with the PMDS layer as a stretchable substrate. Figure 18a shows different color patterns formed with PVP-Ir-PDMS trilayer material with and without red dye. With this, stretch-testing photographs are also displayed for these materials, showing interesting reversible mechanochromic properties (Figure 18b).

However, not just optical changes could be produced by applying external forces. Piezoresponse materials are also commonly utilized for sensing purposes; for example, Jang, Kim, and Lee et al. [74] describe the fabrication of a pressure sensor that posses a large area of microstructured elastomer through a simple and low-cost process, allowing to create a capacitive device with a flexible structure. Experimentally, a soft dielectric Ecoflex film was pre-stretched, followed by UVO exposure. Next, wrinkles on the order of 10 μm were formed on the micro-cut surfaces of the film after stress releases. Finally, electrodes were made from the previously wrinkled film by placing an Au-coated silicon wafer (rigid) and a PDMS (soft) on the top and bottom of the template. Thus, the effect of the films was investigated by comparing the unwrinkled, one-side wrinkled, and two-side wrinkled films on the sensor performance.

When comparing the two-sided wrinkled pressure sensor; with an unwrinkled (smooth surface) one, it improved by 42% and 25% in response and release time. These results minimized the problematic viscoelastic behavior of Ecoflex after the incorporation of wrinkles in the microstructured surface of the sheet, improving its reversible energy release, storage, and sensor performance. A similar example was reported by Xu, Cao, and Lu et al. [75], who fabricated a light-increasing piezoresistive pressure sensor based on a rose petal-templated PDMS. This substrate includes wrinkled nanopatterns on the surface of polypyrrole (PPy)-based films, providing pressure sensitivity, assembling a broad level of pressure detection with low location limit, high sensitivity, fast response/relief, as well as good stability/durability and exceptional pressure detection performance. More importantly, it was possible to achieve a remarkable improvement in sensitivity (70→120 KPa-1, ˂0.5 KPa) and reduce the detection limit (0.88→0.41 Pa). These new pressure sensors have a potential application in optically controlled fields such as soft robotics, optoelectronics, and artificial intelligence due to their control of light and the incorporation of multiscale hierarchical structures.

**Figure 19:**
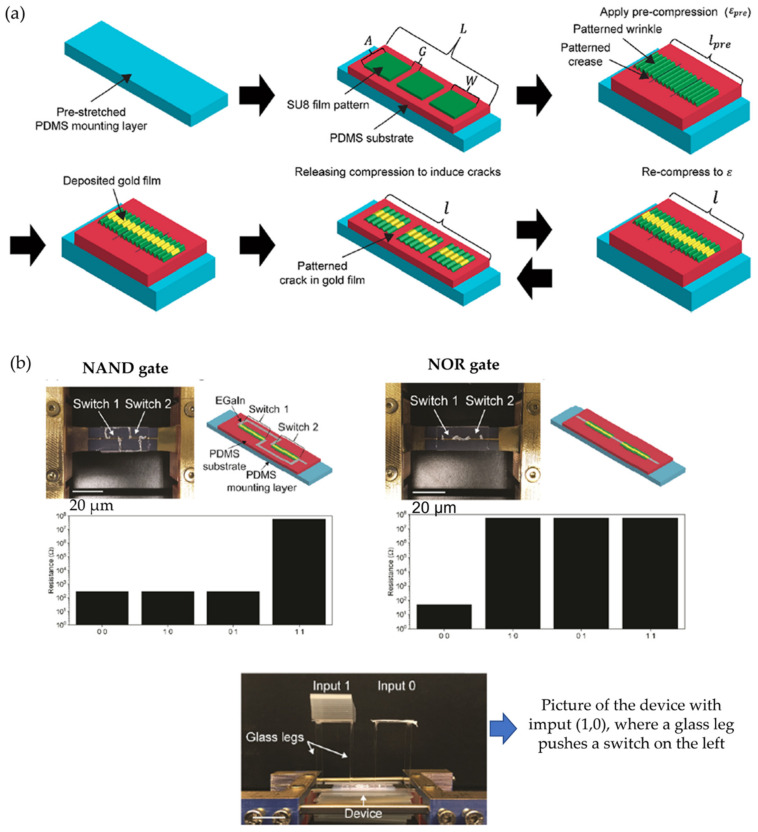
(**a**) Schematic representation of the strategy used to fabricate switchable surfaces. (**b**) Mechanical logic gates (NAND gates, NOR gates). The final image of the device is presented with an input of (1,0), where a glass leg pushes a switch to the left. Reproduced with permission from reference [76]. Copyright 2020, American Chemical Society (ACS).

Finally, it is worth mentioning that there are some other types of mechanic responses, like the one exposed by Hayward and Ouchi [76], that demonstrated a method in which surface deformations such as wrinkles, creases, and cracks are based on three modes to manufacture stamped surfaces with interchangeable electronic properties. Thus, the regions where the wrinkles are providing stretchability and flexibility. Simultaneously, the patterns were molded into the conductive films’ long and straight cracks, presenting large and tunable cracks between one hundred micrometers and centimeters (Figure 19a). Using this method, the authors overcome the limitations of previous switches through mechanically activated electrical switches with adjustable switching voltages from 0.05 to 0.18 and an on/off ratio of high than 10^7^. Figure 19b shows the connection of the two patterns in parallel (NAND gate) and series (NOR gate) in the different devices. As a result, they offer an attractive application in the stretch and pressure-sensitive shielding potentials and coatings because the devices respond to in-plane and out-of-plane forces/deformations.

**Figure 20:**
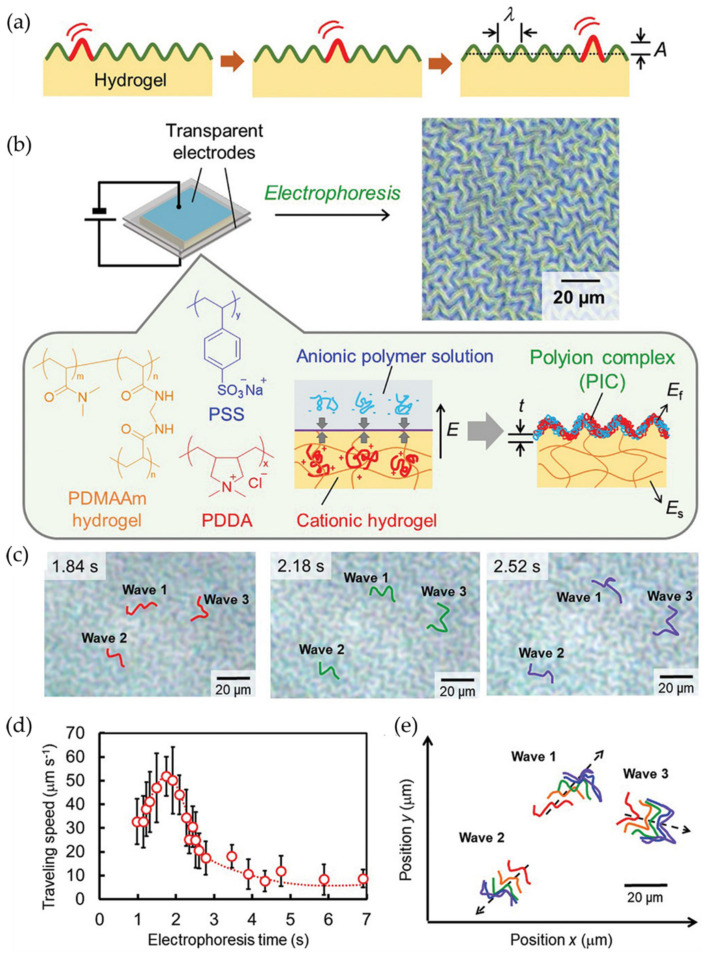
(**a**) Representative diagram of the generation of traveling waves of wrinkles on the hydrogel surface, (**b**) process of wrinkling of the hydrogel surface by electrophoresis, (**c**) images of the wrinkling process on the hydrogel surface during electrophoresis, (**d**) graph of the displacement velocity as a function of electrophoresis time and (**e**) Trajectories of three different waves tracked in the surface. Reproduced with permission from reference [77]. Copyright 2022, John Wiley & Sons, Inc.

Similarly, Kashihara et al. [77] fabricated a polyion complex (PIC) in a hydrogel layer through the electrophoresis process, observing the formation of wrinkled patterns using an electric field stimulus that allows wrinkle formation. The representative scheme of Figure 20a explains the wave generation process in the wrinkled areas of the polymer. Through the electrophoresis process, an anionic polymer poly(4-styrenesulfonate) (PSS) was deposited on the surface of a cationic polymer (PDMAAm) and connected between two electrodes. By administrating an electric field, a wrinkled pattern was formed on the material’s surface through the cationic gel functioning as an anode and the anionic aqueous solution as a cathode; these patterns are formed due to the voltage mismatch in the PIC layer (Figure 20b). Figure 20c shows the wrinkled patterns obtained after applying the electric field, determining that the wrinkles formed remained static. Still, they returned to their dynamic state when exposed to the electric field. Figure 20d shows the displacement of wrinkles on the material’s surface as a function of the speed and time of the electrophoresis process. It was determined that when the first wrinkles have formed, the displacement speed gradually increases, reaching a maximum of ~50 μm/s at 2 s. While higher the electric field, the faster the wrinkles move (increasing speed). Figure 20e shows the trajectories of the wrinkles during the movement provoked in the electrophoresis process.

**Figure 21:**
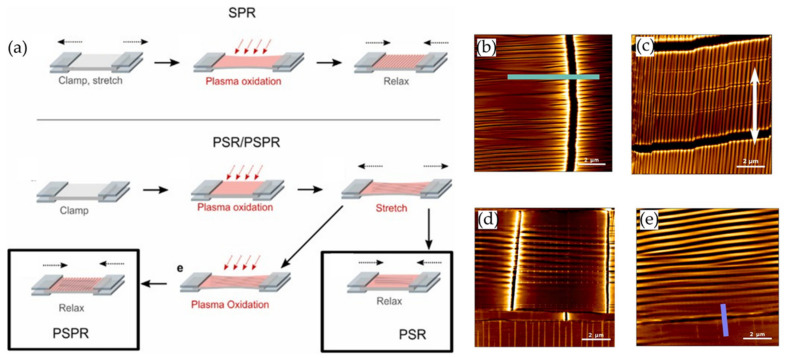
(**a**) PDMS wrinkle manufacturing process, micrographs of wrinkles, (**b**) SPR, (**c**) PS, (**d**) PSR, and (**e**) PSPR. Reproduced with permission from reference [78]. Copyright 2022, Elsevier Ltd.

Similarly, Wawryk et al. [78] fabricated wrinkled patterns in PDMS using a multi-step procedure. Figure 21a shows a representative scheme of the different manufacturing processes used to create the wrinkled surfaces. The PDMS films were attached to two metal plates, one static and the other in motion, allowing uniaxial movement (stretching up to 16%). Then the films were exposed to air plasma with a power of 30 W for 5 min, followed by a cooling process (1 min) to relax it finally. This series of procedures generate wrinkled patterns on the surface of the material. Atomic force microscopy (AFM) was used to analyze the wrinkled patterns obtained in each one of the different fabrication steps to form the wrinkles (Figure 21b–e). The other surface structures depicted in Figure 21b–e correspond to varying combinations of the processes used to create wrinkled patterns (air plasma (P), strain (S), and relaxation (R)).

In Table 4, it is possible to observe a summary of the examples mentioned in Section 2.5 for mechanical-sensitive materials used to create smart wrinkled patterns. The table also depicts the methodology used to fabricate the wrinkles and its possible application.

### 2.6. Chemical Reaction/Gas/Solvent Response

The most simple and common way to fabricate wrinkled patterns is via swelling/deswelling processes; for example, Damman and Vandeparre [79] proposed a mechanism to link molecular diffusion (solvent diffusion) and the stability of a rigid membrane attached to a polymeric layer to generate wrinkled patterns. They produced films with thicknesses between 0.1 and 1 μm made up of a polystyrene (PS) system deposited on a bare silicon (SiOx) substrate. Subsequently, using thermal evaporation, thin layers of titanium (Ti) with a thickness of 10 to 20 nm were deposited on the polymer surface. The multilayer was then immersed in toluene vapors at room temperature to produce wrinkles. These created geometric patterns comprised various fold formats (herringbone, parallel or radial stripes, among others) and dynamically different ones (continuous or discrete) from the diffusion and minimization of wrinkle energy. This work is relevant because it allows them to understand the instability of the different morphological patterns and the formation or elimination of topological imperfections.

Other methodologies, like the one reported by Oscurato et al. [80], involve innovative procedures to spontaneously produce wrinkled surfaces due to naturally occurring chemical transformation processes. The procedure is carried out in smooth thin solid films of 5,6-dihydroxyindole (DHI) over 30 to 60 days in ambient conditions. Due to gradual oxidation in the film, shear stresses were generated in the material inner layers, thus producing a slow swelling/expansion on the material followed by a fast stiffening of the upper layer, exposed to higher oxygen levels. Interestingly, this phenomenon is only observed with layer thicknesses over 250 nm.

However, there are also more intriguing methods to produce and control wrinkles on the surface of the materials; for example, Corté et al. [81] reported a methodology based on the halogenation of rubber to increase the glass transition temperature within a thin layer on its surface that allows creating reversible wrinkled patterns. Thus, the effect of surface bromination of polychloroprene-based rubber films was investigated by examining three different aspects: (1) the spatial distribution of bromine after immersion of a rubber film in a bromine solution, (2) how the glass transition of the material surface is modified by bromine, and (3) how the effect of temperature and mechanical stress are sensitized. Microscopic wrinkling occurs at temperatures below the brominated layer’s glass transition, which was visualized in situ in the stretched and unstretched films in the thermomechanical experiments. When the wrinkled layer is submitted to higher temperatures, the wrinkles are eliminated, and it recovers its original magnitudes. This methodology is highly attractive due to its ability to automate surface patterning techniques or design a thermosensitive surface roughness using the gradient of bromine content and the thickness of the modified layer.

Similarly, Zhang et al. [82] report the hysteresis effect produced by cyclic heating and cooling graphene sheets giving rise to reversible wrinkling patterns. The system was constituted by monolayer graphene structure growth on the Pt(111) surface via CVD or surface aggregation. In an ultrahigh vacuum, the substrate was cleaned by Ar+ sputtering, oxidation, and annealing cycles. The surface was observed by low-energy electron microscopy (LEEM). It was found that the oxidative process of graphene and CO was generated underneath the graphene layers. These results suggest that the wrinkles formed may interact as nanometer-sized gas pockets and drive chemical reactions in the graphene structure.

Following the same topic, Jiang et al. [83] reported a simple and effective way to design reversible wrinkle patterns that, through the Diels-Alder (D-A) dynamic chemical reaction, can control wrinkle removal and adjustment in situ. The furan-grated bismaleimide (BMI) and polybutyl acrylate (FBA) top layer system is coated onto the PDMS elastomer. Wrinkle formation was then induced through hardening and crosslinking with a high Young’s modulus of the top layer product of the D-A reaction, releasing localized stress to minimize total system energy. Afterward, to produce the decrosslinking of the FBA/BMI top layer, causing wrinkles to disappear and the surface to become smooth, the sample was heated at 120 °C for 20 min to cause a retro-D-A reaction. This mechanism based on D-A reactions provides high versatility in obtaining highly reversible crinkle patterns, providing precise control over characteristics such as wettability, adhesion, and optical properties.

**Figure 22:**
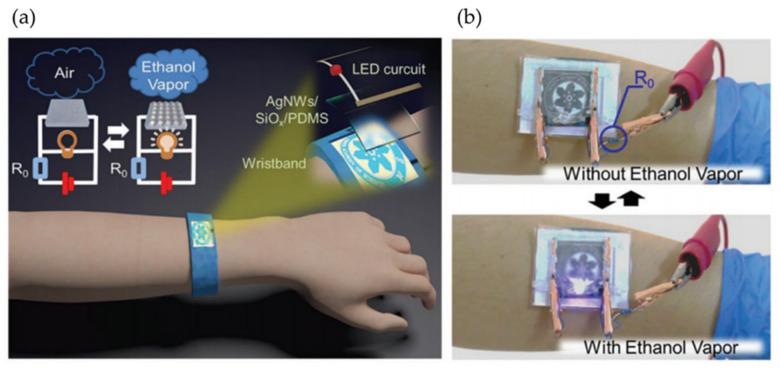
(**a**) Schematic illustration of the smart wearable and flexible electronic device based on the Ag nanowire/PDMS multilayered film and (**b**) a proof-of-concept wearable device that responds to ethanol vapor directly dynamic visual and electrical feedback. Reproduced with permission from reference [84]. Copyright 2019, John Wiley & Sons, Inc.

Recently, Qu et al. [84] developed a method for fabricating wrinkled patterns over multilayered materials based on PDMS/Ag nanowire films. Interestingly, wrinkled structures can be formed dynamically in response to volatile organic compounds (such as ethanol, toluene, acetone, formaldehyde, and methanol) due to the multilayer-induced instability caused by their different swelling capabilities. Its multilayered condition could be helpful for responsive and flexible sensor applications as materials present a switchable dual-signal response (transparency and resistance). By controlling PDMS’s modulus, composition, and layer thickness, tunable transparency and resistivity could be obtained. As a result, the film with a UVO treatment time of 14 min and a base-to-curing agent ratio of 60:1 can achieve the most sensitive transparency and resistance signal in response to ethanol vapor of different concentrations. The multilayered films also show more selectivity toward ethanol vapor than other volatile organic compounds. Figure 22 shows a schematic description of a wearable device design as a dual-signal sensor that reacts to ethanol vapor.

**Figure 23:**
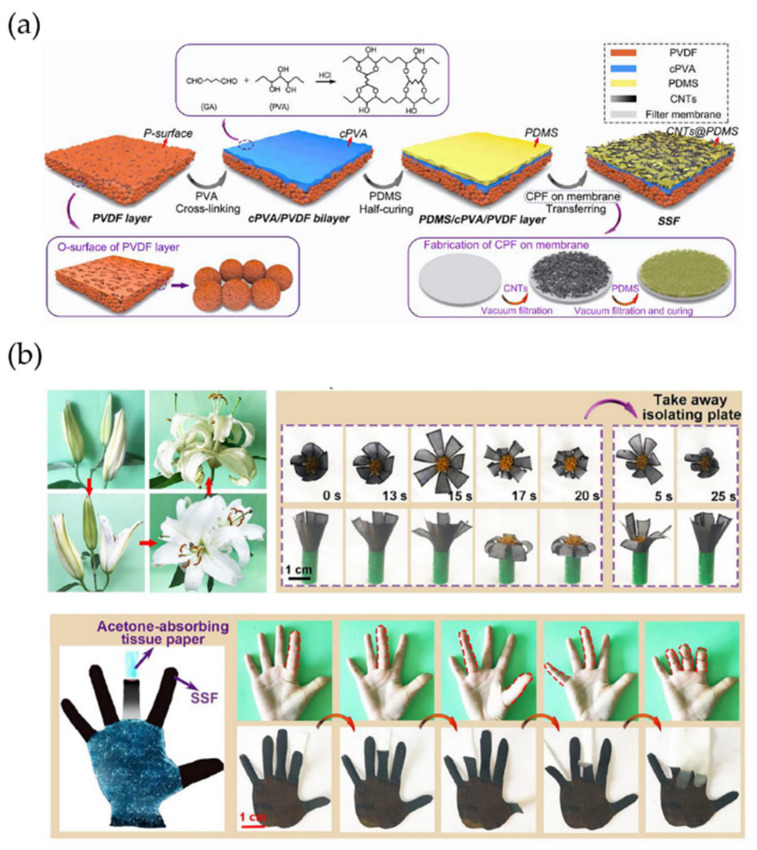
(**a**) Schematic diagram indicating the fabrication of durable stimuli-responsive superhydrophobic films (SSF). (**b**) Illustration of the flower and finger devices. Reproduced with permission from the reference. Reproduced with permission from reference [85]. Copyright 2020, Elsevier Ltd.

Similarly, Li and Zeng et al. [85] fabricate functional superhydrophobic materials with stimuli-responsive characteristics. They manufacture these devices by integrating a three-layer structure, a poly(vinylidene fluoride) (PVDF) layer located between two poly(vinyl alcohol) (PVA) layers. The first one tends to absorb solvent vapors to induce swelling, and the second one repulses solvent vapors with finality to maintain their original dimensions (Figure 23a). The samples exhibited superhydrophobicity with a high contact angle of 163° and a low sliding angle of 4.5°. Figure 23b illustrates the potential applications, such as a biomimetic flower, which respond to the absorption/desorption of acetone vapor. Like lilies in nature, the biomimetic glower displayed a dynamic blooming and fading process. Another example was designed as five fingers and assembled into a hand-patterned actuator. Vimineous acetone-absorbing tissue papers were placed under the fingers to activate their bending at ambient temperature.

Crosby and Kim [86] fabricated stimuli-responsive surfaces based on osmotically driven wrinkling with hierarchical morphology and controlled wavelength. These studies have been based on the PDMS layer’s oxidation using UVO supported over the silicon substrate. Subsequently, an ethanol droplet was placed on the oxidized PDMS surface, and wrinkles formed spontaneously, confined within the droplet’s trace, and then disappeared upon evaporation of ethanol. Other volatile solvents, such as alcohols, THF, and pyridine, could also achieve this response, affecting the wrinkles’ wavelength and amplitude due to their different swelling properties. It can also control the wrinkles patterns’ dimension and morphology by changing the oxidation time and the soft layer’s thickness. This versatility method allowed the performance of specific functional devices, such as reversible channels, tunable microlens arrays, and facile printing of nanoparticle assemblies [87].

## 3. Non-Planar Wrinkled Structures

In general, most of the research conducted in recent years by the scientific community has been related to the fabrication and application of wrinkled patterns on flat surfaces. However, numerous applications often tend to be on non-planar surfaces, so the generation and fabrication of these surfaces can sometimes be challenging. Several research groups have studied the experimental and theoretical mechanical framework, models, and constitutive relationships involved in generating non-flat wrinkled patterns to predict the final shape accurately and pattern distribution [88,89,90]. This section will discuss the most exciting studies on fabricating wrinkled patterns on non-planar surfaces. To organize this section, we will divide it into different topics according to the type of non-planar structure, such as solid core spheres, solid core cylinders, hollow spheres, and cylindrical tubes.

### 3.1. Solid Core-Shell Spheres

Several of these non-planar wrinkled patterns can modify their adhesion with different surfaces depending on the type of micropattern generated on their surface. Controlled adhesion is essential for developing these materials in many applications, from micro/nanoelectronic systems to bioimplants. Therefore, incorporating geometric features/patterns on the non-planar surface has interested several research groups in recent years. Crosby et al. [91] studied the adhesion of spherical objects with topographical surface patterns created by cover wrinkling/deformation techniques. The surface modifications were fabricated on PDMS lenses with a curvature (R) radius of 7 mm. Surface adhesion was quantified as a function of wrinkle dimensions such as wavelength (20 to 70 μm) and amplitude (0.3 to 5.0 μm). The applied deformation is regulated by varying the vapor pressure and swelling solvents. Ethanol and isopropanol have been used as swelling solvents, and the vapor pressure of these solvents is controlled by mixing glycerol in different proportions.

On the other hand, they describe the effect of adhesion force as a function of wrinkle morphology (wavelength and amplitude) and the dependence of the cumulative effect on the radius of curvature of the surface. Jagota and Yang et al. [92] studied the adhesion of a one-dimensional (aligned) PDMS wrinkled surface using a spherical glass probe. In this study, it was observed that adhesion tends to decrease with increasing wrinkle width. Similarly, Feng et al. [93] reported surface buckling and the morphological transition from a soft-core sphere using a light-tension volumetric growth theory. In addition, they showed that the critical buckling condition and the induced patterns were susceptible to the thickness of the sphere shell and the material’s mechanical properties. As mentioned in the introduction section, the increased available surface area due to wrinkling improves several properties related to particles’ contact area. For example, the conductive layer with non-planar morphologies becomes more sensitive than planar films. However, ultrasensitive pressure sensors assembled using non-flat conductive wrinkles are rare due to the lack of a wavy arrangement with noticeable surface buckling and adequate adhesion on soft substrates. Wang et al. [94] proposed the formation of wrinkles with electrically conductive behavior by combining polymer swelling and electrodeless deposition. Thus, plasma-treated PDMS films were adsorbed onto a bovine serum albumin layer, followed by swelling in chloroform. A silver film was placed on the swollen PDMS after electrodeless deposition in a mixture of Tollen’s reagents. The pressure sensor was fabricated by superimposing two face-to-face wrinkled surfaces, forming a piezoresistive sensor. In this study, the resistance change attributed to the conformal contact change between two conductive layers is more noticeable for wrinkles with longer wavelength and amplitude, which is described as better pressure sensing performance. Thus, the sensor fabricated with silver wrinkles on the patterned pillar was extended to sound-sensing applications.

Similarly, Zhang et al. [95] synthesized core-shelled composite microspheres with a wrinkled micro-structured carbon-titanium dioxide (C/TiO_2_) created by taking advantage of microwave absorption properties. Their group established a three-step method to manufacture these wrinkled spheres, based on the polymerization of polyglycidyl methacrylate/divinylbenzene polymer spheres (PGMA/PDVB), a TiO_2_ coating of the spheres mediated by hydrolysis of tetrabutyl titanate (TBT) and vacuum carbonization of PGMA/PDVB/TiO_2_, forming C/TiO_2_ composite microspheres. It is worth mentioning that this manufacturing configuration allows controlled regulation of the polymerized wrinkled spheres, their electromagnetic parameters, and their superficial wave reflections.

Other novel applications using wrinkled non-planar (spherical) surfaces are, for example, the one reported by Cao et al. [96], who developed a novel functional interlayer by wrapping dispersed zinc sulfide (ZnS) nanospheres and an ultrathin wrinkled carbon film (ZnS/WCF). Specifically, the wrinkled structure of the porous WCF provides sufficient intermediate spaces to hold the electrolyte and ensure the distribution of lithium ions. In addition, ZnS nanospheres with WCF treatment act as anchor sites for LiPSs (soluble lithium polysulfides) entrapment and promote the breakdown of solid Li_2_S with a relatively low surface migration barrier. This composite structure could pave the way for a broad application of Li-S batteries.

**Figure 24:**
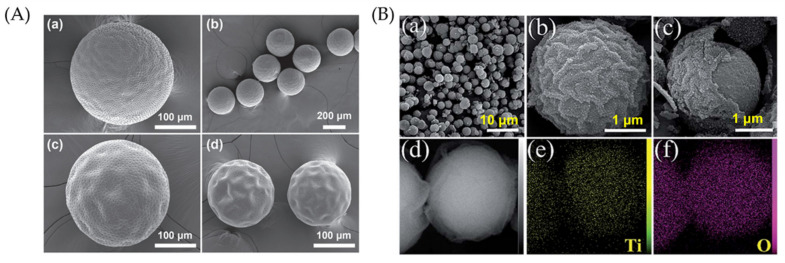
(**A**) SEM images of the dual-wrinkled microparticles from (a,b) PLGA/PDLA-b-mPEG blend and (c,d) PLGA/PCL-b-PEG-b-PCL blend. Reproduced with permission from reference [97]. Copyright 2018, Royal Society of Chemistry (RSC), and (**B**) FE-SEM (a–c), BSE-SEM (d), EDS mapping (e,f) images of the Y@HWS-TiO_2._ Reproduced with permission from reference [98]. Copyright 2021, Elsevier Ltd.

On the other hand, Im et al. [97] fabricate a labyrinthine pattern onto uniform-sized microparticles based on a biodegradable polymeric blend using an emulsion-solvent evaporation method. By changing the particle size, the wrinkled patterns switch from a simple labyrinth morphology to a bi-phase pattern where different wrinkle morphologies coexist. The microparticles were fabricated in a tube-type microfluidic device using various amphiphilic block copolymers based on polycaprolactone (PCL), poly(ethylene glycol) (PEG), and poly(D, L-lactide) (PDLA). These copolymers were dissolved in dichloromethane to obtain an oily solution in which polymer microparticles were formed. By regulating the composition and the sizes of the particles, different non-planar wrinkled structures could be obtained. Figure 24 shows some bi-phase wrinkled patterns formed over the spherical microparticles.

Recently, Peymanfar et al. [99] synthesized a wrinkled Ni nanosheet/grapy carbon microsphere (CMS) nanocomposite. The wrinkled Ni nanostructure was prepared using the co-precipitation and hydrothermal method. Similarly, Li et al. also fabricate CMS structures but with some modifications [100]. PS or PVDF was first dissolved in DMF to fabricate the nanocomposite. Next, CMS, Ni nanosheets, and Ni/CMS nanocomposites with 50 wt% (filler/filler + polymer matrix) were separately suspended in the solution using an ultrasonic bath, then heated and molded into rectangular shapes to investigate the microwave frequency absorption properties of the nanocomposites.

**Figure 25:**
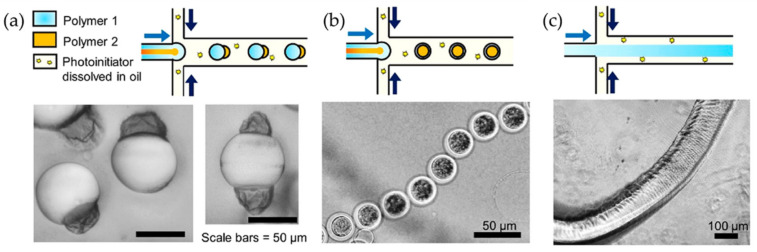
Wrinkled surface patterning formation on various microstructures created using a microfluidic system. (**a**) Janus particles with wrinkled sides, (**b**) Wrinkled core-shell with inner core underneath another hydrogel layer, and (**c**) Wrinkled surfaced fibers. Reproduced with permission from reference [56]. Copyright 2021, American Chemical Society (ACS).

In particular, the results manifest that the architecture’s microwave absorption and shielding structures illustrated considerable reflection loss, efficient bandwidth, and shielding properties with thin thickness due to the wrinkled morphology of Ni and applied media. An additional novel wrinkle manufacturing method was explored by Jeong et al. [56], using oil-water interphase to fabricate the pattern. The appearance of wrinkles is achieved by the dispersion of the photoinitiator suspended in the oil phase on the aqueous phase containing a prepolymer solution. The explained methodology still requires UV exposure to establish a polymerization. In addition, this oil-water generates tension that promotes the formation of wrinkles on curved structures such as solid spheres or cylinders. Figure 25 describes a schematic representation of this one-step wrinkle-forming procedure using curved surfaces.

### 3.2. Solid Core-Shell Cylinders

Another type of structure commonly used to fabricate non-planar wrinkled surfaces is cylinders; for example, Cao and Feng et al. [101] investigated the phenomenon of wrinkle formation on the surface of an elastic substrate subjected to in-plane compression. Thus, two theoretical and finite element analyses of the variations of the substrate modulus along the depth direction (exponential functional and power function) were performed. Feng et al. [102] performed theoretical analysis and numerical simulation to study the surface wrinkling and patterns evolution of cylindrical elastomers with core-shell structure induced by material swelling/shrinkage. Their studies showed that a slight compression on the shell (the shell is supposed to be stiffer than the core) could generate the first buckling characterized by sinusoidal patterns.

**Figure 26:**
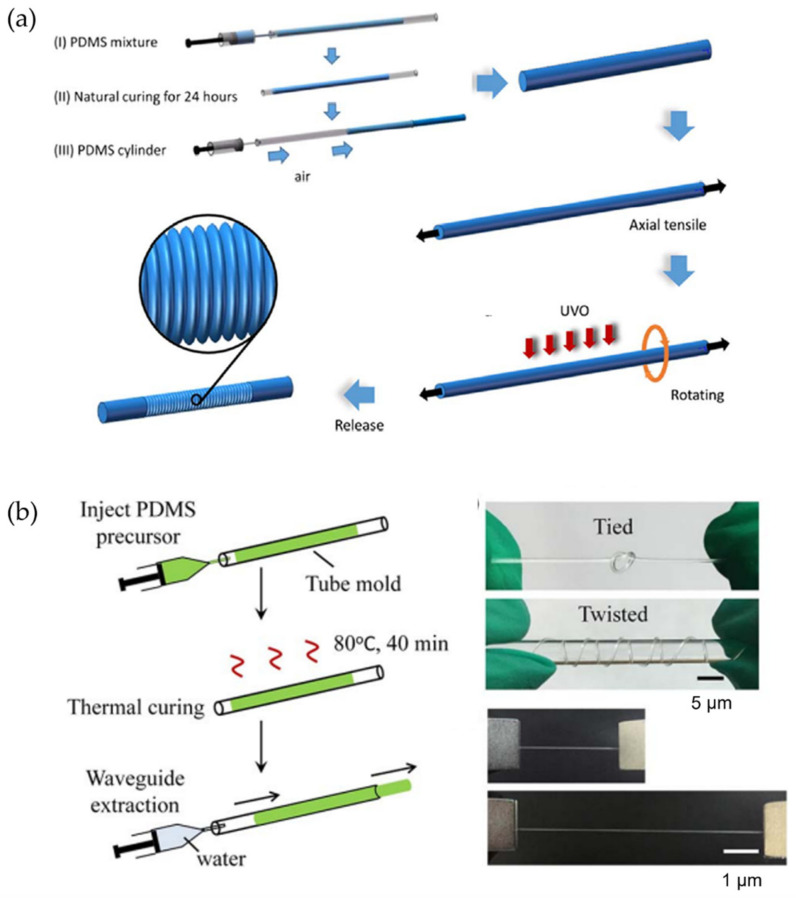
(**a**) Wrinkled PDMS cylinder preparation process and (**b**) fabrication and testing of the mechanical flexibility of the PDMS fibers. Reproduced with permission from references [103,106]. Copyright 2018, American Physical Society. Copyright 2019, Elsevier Ltd.

Further pressure on its surface can lead to a transformation from wrinkles to folds, resulting in the formation of hairpin patterns that double the first buckling period. The surface modifications that occur at the first and second bifurcation can be adjusted by modifying the mechanical and geometrical properties of the structure. In 2019, Wang et al. [103] investigated a novel method that created concentrical wrinkles on PDMS. The application of UV/O was carried out on pre-stretched PDMS cylinders in constant rotation, which resulted in the formation of annular wrinkles upon release (Figure 26a). Through simulation and experiments, pre-stretching (mechanical strength and stretchability) and exposure time were related to wavelength and wrinkle amplitude. Pearce et al. reported on the surface wrinkling of a thin elastic film attached to a sizeable adjustable cylinder from the perspective of large strain elasticity, concluding that curvature delays the instability of wrinkles [104]. Their findings confirmed the simulation results of Patricio et al. [105], which state that the orientation of the wrinkles can be longitudinal (along the fiber axis), polar (along the fiber perimeter), or a combination of both, depending on the mechanical and geometrical parameters of the fiber. In 2018, Yang et al. developed a highly flexible and stretchable optical strain sensor [106]. In this research line, doped and undoped PDMS was fabricated. Dye molecules were added to the PDMS precursor before curing to dop the PDMS. Thus, the undoped sample showed high transparency, while the PDMS doped with rhodamine B dyes showed higher attenuation, which is attributed to absorption and scattering due to the spatial inhomogeneity of the doped dyes.

On the other hand, PDMS fibers showed excellent mechanical flexibility so that they could be easily bundled and twisted (Figure 26b). The mechanical test responses (dynamic and static) showed stable, reliable, and repeatable performance. The sensor demonstrated that it could detect deformations over a wide dynamic range of 100% with an accuracy of ± 0.91%. An example was used to detect dynamic body movements, such as breathing and joint movement, all performed in real-time.

### 3.3. Hollow Spheres

However, not just solid structures were developed to form wrinkled patterns over non-planar surfaces, Zhu and Xing et al. [107] synthesized a novel chitosan material (ECS/Ca/CTA) to adsorb SDBS (sodium dodecylbenzene sulfonate) with high efficiency by using hollowed-out spheres with wrinkled patterns on their surface. Glutaraldehyde (GA) was used to crosslink CS (chitosan) into a wrinkled hollow sphere, which increases the specific surface area, chemical stability, and capability of capturing SDBS. The adsorption procedure fits well with the pseudo-second-order kinetic mechanism, and the adsorption equilibrium experiment fits the Langmuir model. A similar example was the pleated hollow-sphere material (CS/Ca/CTA) mixing CS and CaCl_2_ in acetic acid solution followed by cross-linking with GA and quaternized using (3-chloro-2 hydroxypropyl) trimethylammonium chloride (CTA), which efficiently adsorbed SDBS [108].

Following the same trend, Badiei and Luque et al. [98] reported the fabrication of advanced hydrogenated yolk/wrinkle structures on TiO_2_ shell (Y/HWS-TiO_2_); these materials demonstrated excellent photocatalytic activity under visible light irradiation for the selective oxidation of alcohols at room temperature. The synthesis includes sequential processing steps, such as solvothermal reactions using polyethylene glycol as soft templates, partial etching, and hydrogen treatment. This research can prove the elaboration of a randomly assembled wrinkled surface on the microsphere’s surface (solid core). The photocatalytic oxidation activity of the materials was tested in the selective oxidation reaction of benzyl alcohol to benzaldehyde, obtaining 90% conversion and 97% selectivity. The results showed that these particles possess high porosity and hollow space in the yolk/shell structure, which is beneficial for molecule displacement and attachment, along with a thin, wrinkled shell, which can promote molecule dispersion and create active sites exposed to reagents. In a similar example provided by the same studies, Zeng et al. [109] synthesized hierarchical CoFe_2_O_4_ double-shelled hollow spheres (DHSs) with a wrinkled porous surface. This structure exhibited high sensitivity to ammonia (NH_3_) with good stability and fast response-recovery capability at the optimal operating temperature (240 °C), an effect mainly related to two characteristics: its sizeable specific area and, secondly, its penetrable hierarchical shells.

On the other hand, Zhao et al. [110] prepared a hollow tremella-like graphene sphere/tin dioxide (HTGS/SnO_2_) composite via emulsification and impregnation. This structure (HTGS/SnO_2_) presents excellent electrochemical stability. The graphene sphere’s hollow structure allows the simultaneous insertion of lithium ions from the inner and outer surfaces. Meanwhile, the tin dioxide particles are uniformly dispersed by the graphene’s surface’s wrinkles, thereby enlarging the space for the volume expansion of tin dioxide to avoid contact with the electrolyte. As a result, the initial discharge capacity reached ~1760 mAh g^−1^, and the reversible capacity was ~1170 mAh g^−1^. After 50 cycles, the reversible capacity was maintained at ~940 mAh g^−1^, and the Coulomb efficiency was consistently above 96%.

Similarly, Davarani et al. [111] have constructed a supercapacitor (GW-MSNGOHS//GW-YS-NFOHS) consisting of an electrode with a high porous nanoarchitecture and superior energy density. It consists of a multilayered NiGa_2_O_4_ hollow sphere wrapped in graphene (GW-MSNGOHS) with an exceptional surface area of ~115 m^2^ g^−1^ as a new positive electrode material and a NiFe_2_O_4_ hollow sphere with graphene shell (GW-YS-NFOHS) as a negative electrode material. In 2020, Davarini and Zardkhoshoui [112] encapsulated multi-shelled zinc-cobalt selenide hollow nanospheres as positive electrodes and yolk-double shell cobalt-iron selenide hollow nanospheres as negative electrodes into graphene networks a self-templating technique. The hollow nanospheres, called G/MSZCS-HS and G/YDSCFS-HS, present high electrical conductivity and unique structures, showing exceptional positive and negative capacities (~377 mAh g^−1^ and ~293 mAh g^−1^), superior rate performances, and robustness after 12,000 cycles.

**Figure 27:**
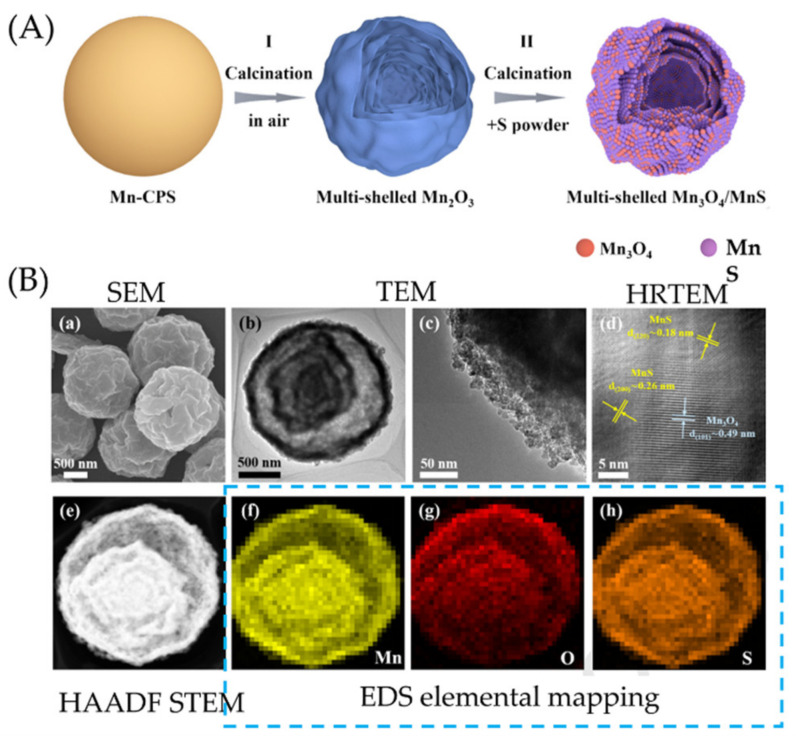
(**A**) Schematic illustration for the fabrication of Mn_3_O_4_/MnS and (**B**) (a) SEM image, (b,c) TEM image and (d) HRTEM image of Mn3O4/MnS heterostructures building multi-shelled hollow microspheres; (e–h) HAADF STEM and EDS elemental mappings of different elements of Mn, O and S recorded from a single heterogeneous Mn3O4/MnS multi-shelled hollow sphere. Reproduced with permission from reference [113]. Copyright 2022, Elsevier Ltd.

Likewise, Qi et al. [113] proposed a supercapacitor (electrochemical capacitor) composed of Mn_3_O_4_/MnS heterostructure building multi-shelled hollow microspheres and active carbon as the cathode and anode, respectively. Figure 27a shows the synthetic route; first, Mn-based coordination polymer spheres (Mn-CPSs) can be synthesized by the solvothermal reaction of Mn^2+^ ions with organic compounds. The multi-shelled Mn_2_O_3_ spheres were then prepared through a pyrolysis process of the Mn-CPSs (step 1). Posteriorly, it is reacted with sulfur power, and heterogenous Mn_3_O_4_/MnS multi-shelled hollow spheres are obtained (step 2). The morphology was explored through SEM, TEM, and HRTEM, showing a higher wrinkled degree than the multi-shelled Mn_2_O_3_ hollow (Figure 27b). The devices exhibited a high capacitance of ~185 F g^−1^ at 1 A g^−1^, with substantial energy density (~66 W h kg^−1^) and power density (16,000 W kg^−1^). The superior electrochemical performances could be attributed to the heterostructures and the stable multi-shelled structure.

A recent implementation for hollow wrinkled spheres was proposed by Wang et al. using SnO_2_ hollow microspheres with Au-Ag bimetallic nanoparticles for triethylamine gas detection. This configuration showed high responses, fast response/recovery speed, and long-term stability. These wrinkled spheres were prepared through combinations of Sn_4_^+^ and resorcinol-formaldehyde (RF) resin polymer spheres, followed by a calcination process [114].

**Figure 28:**
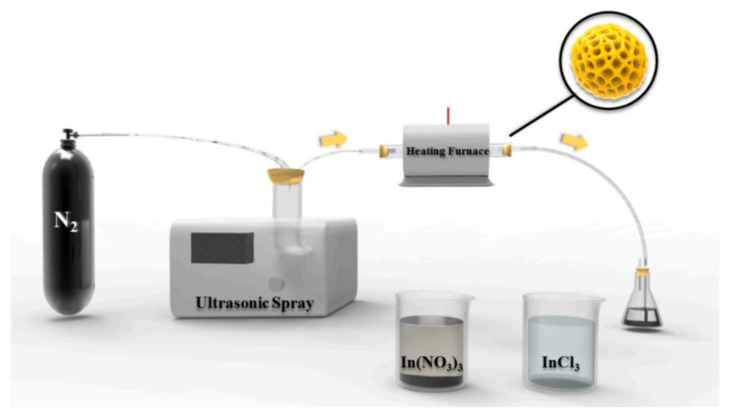
Schematic representation of the experimental procedure followed to fabricate ultrasonic pyrolysis spray-based wrinkled microspheres. Reproduced with permission from reference [115]. Copyright 2021, Elsevier Ltd.

With a similar objective, Li et al. [115] implemented a spray pyrolysis methodology to manufacture wrinkled microspheres for high-performance ethanol sensors. This group studied wrinkled In_2_O_3_ microspheres, finding a short recovery time, high sensitivity, and up to 100 ppm ethanol detection with a high dependence on atmospheric humidity. Figure 28 presents a schematic description of the process followed in the pyrolysis of wrinkled In_2_O_3_ microspheres.

Another exciting application of wrinkled hollow spheres is the use of transition metal oxides as energy storage for future innovations in electronic devices. One of the research related to this feature is that of Naffakh-Moosavy et al., in which the morphological and electrochemical properties of V-doped Co_3_O_4_ hollow spheres (MS-V-CO) produced by solvothermal/restoration were studied. The fabricated material resulted in low internal resistance, fast kinetics, remarkable reversibility, acceptable capacitance, and extremely high available surface area, which is a viable candidate for implementing a modern capacitor to be used in the future [116].

Finally, Moreno and Carp et al. [117] synthesized an open hollow structure of Co_3_O_4_ by a three-step procedure, a green alternative to the Pechini method that employs a carbohydrate as a polymerization agent. First comes the initial formation of Co^+2^ (citrus soluble coordination compounds), and the second part comes the hydrothermal processing at (180 °C) in fructose, followed by thermal air processing of the obtained precursors. Notably, the obtained Co_3_O_4_ consists of crystallites with sizes ranging from ∼26 nm in concave hollow shapes with multilayered spheres, which have filigree and wrinkled walls. The lack of citric acid allowed for hollow double-shell morphologies with denser walls. On the other hand, the presence of citric acid imparts high specific capacity, rate performance, and cycling stability after 250 cycles to the structures, compared to the same hollow multilayered spheres synthesized in one ambient in the absence of citric acid. From a broader point of view, the research is an excellent example of how simple synthetic procedures using neither raw materials nor sophisticated equipment can offer feasible access to elegant, elaborate, and high-performance anode nanostructures for lithium-ion batteries.

### 3.4. Cylindrical Tubes

Cylindrical tubes are the last type of non-planar wrinkled structure in this review. For example, Geng et al. [118] have designed a novel microscale fabrication technique based on structural self-assembly processes to create periodically controlled ring-shaped disturbances (wrinkled patterns). The results show that the wrinkles are formed by stretching a soft polymer fiber made of PDMS at tensions ranging from 10% to 200%, followed by UV/ozone exposure to generate a hard SiOx film on the soft fiber to release the stress of the fiber after its formation. Geng et al. could identify the main parameters that control wrinkle dimensions and distribution over the polymeric fiber.

Similarly, Yang et al. [119] developed an innovative, simple, low-cost method to fabricate periodic wrinkled patterns over elastomeric substrates. PDMS films were prepared over polyethylene terephthalate (PET) substrates using the spin-coating process. Then, the PDMS-PET system was bent until it reached a specific deformation and was exposed to an oxygen plasma treatment during different periods. The plasma exposition generates SiO_x_ molecules on top of the material, thus producing an oxidized thin layer with increasing stiffness, triggering the formation of a wrinkled pattern after bending release. These materials show good visual performance in transmittance diffraction experiments, enabling the fabrication of highly ordered grating devices, which can further develop portable and cost-effective applications to provide a valuable method for making other optical micro-detection devices.

On the other hand, Sampathkumar et al. [120] fabricate sculpturing wrinkled structures into soft polymers (SU8 photoresist and PDMS) by thermal treatment of graphene flakes. The wrinkles were formed via biaxial compressive stress to the graphene flakes controlling graphene/polymer composites’ heating. The wrinkled pattern dimensions are directly related to the graphene’s thickness and the young modulus of the polymer substrate. On the other hand, the orientation of the pattern to the shape of the flake. This fabrication process also serves as a simple route to pattern the surface of soft polymers with wrinkling wavelengths that can be as low as ~50 nm.

**Figure 29:**
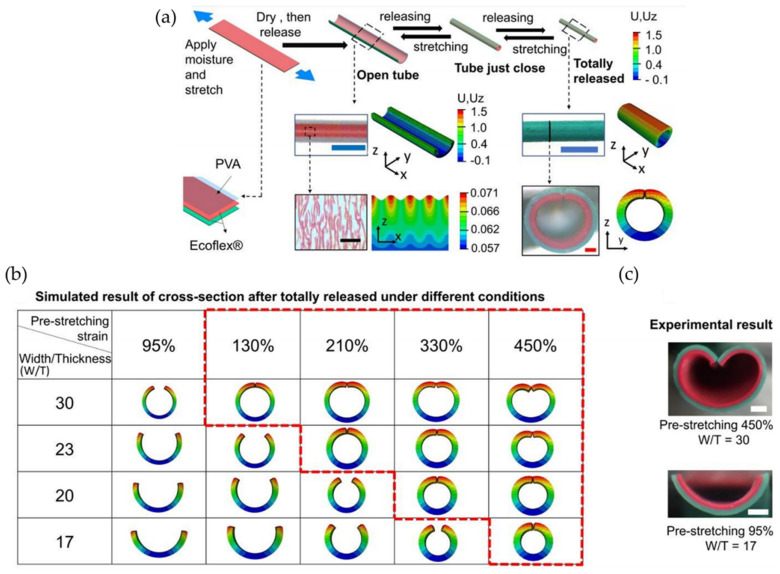
(**a**) Schematic representation of the PVA-Ecoflex^®^ tubular geometry fabrication, (**b**) simulated result, and (**c**) experimental result of the tube cross-section after released under different width/thickness ratios and pre-stretching strains. Reproduced with permission from reference [121]. Copyright 2020, Royal Society of Chemistry (RSC).

Notably, Sun et al. [121] studied the spontaneous formation of wrinkled patterns on tubular structures by using poly(vinyl alcohol) (PVA) and Ecoflex^®^. This bilayered system was exposed to room temperature water mist to plasticize the PVA, followed by immediate stretching to predetermined strain. Then, the bilayered system is dried and released to form the tubular structure. It was possible to predict the elastomeric system’s deformation and the wrinkled pattern morphology and distribution formed on top of the material through finite element analysis. The material presents a strong anisotropic constitutive response based on incompressible hyper-elastic neo-Hookean material. Figure 29a shows a schematic description of the process followed to form the self-wrapping tubular structure. Figure 29b,c offer theoretical and experimental results of the system’s overall tube shape under different strains.

**Figure 30:**
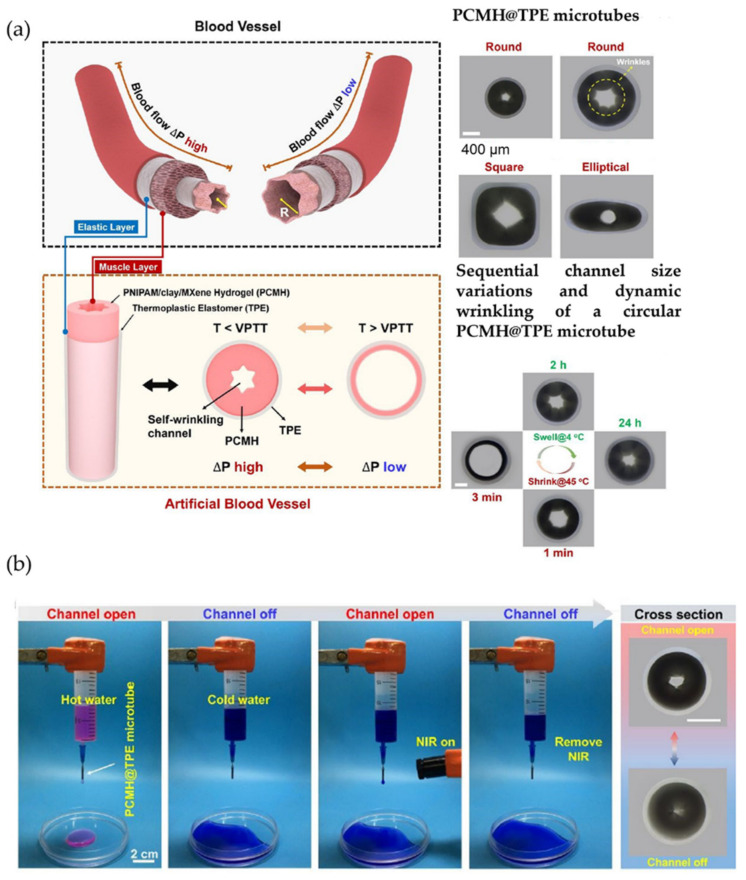
(**a**) Schematic illustration of the layered structure, hydraulic pressure sensing, and actuating functions of artery walls as well as an artificial blood vessel, PCMH@TPE microtube, and (**b**) photographs of PCMH@TPE microtube as a smart microvalve for controlling water flow with the channel open/closed by hot/cold water and re-opened/re-closed by irradiation or remotion of NIR. Reproduced with permission from reference [122]. Copyright 2022, Elsevier Ltd.

Likewise, Sun and Wu et al. [122], following the hollow structure of multilayered blood vessels (lamellar and muscular layers), developed a hydrogel-elastomer hybrid microtube with the quality being elastic and soft. To achieve this, they used a thermoplastic elastomer (TPE) based on a styrene-isoprene-styrene block copolymer as the outer layer and a poly(N-isopropyl acrylamide) (PNIPAM)/clay/Mxene (PCMH) hydrogel which is sensitive to heat and infrared light as the inner layer, as shown in Figure 30a. Due to the mechanical instability presented, the TPE layer has a self-wrinkled inner surface with uniaxial ridges, which is remarkably similar to natural blood vessels. The photothermal effect caused by the NIR is used as a non-contact stimulus model to change the body temperature. It should be noted that the water temperature causes reversible changes in channel size and the appearance/disappearance of microtube wrinkles. Figure 30b a microvalve system connected to a syringe to control the fluid flow, using rhodamine (hot water) and methylene blue (cold water). The channel was opened via NIR irradiation (30 s) due to the rapid contraction of PCMH and then closed for 15 min after the removal of the NIR exposure. In this way, temperature and NIR-induced flow transport and blockage are performed at a distance, such as vasodilatation of the muscle layer and constriction of blood vessels to regulate blood flow. This smart microtubing opens the door to understanding the effect of tissue surface topography on biological functions.

**Figure 31:**
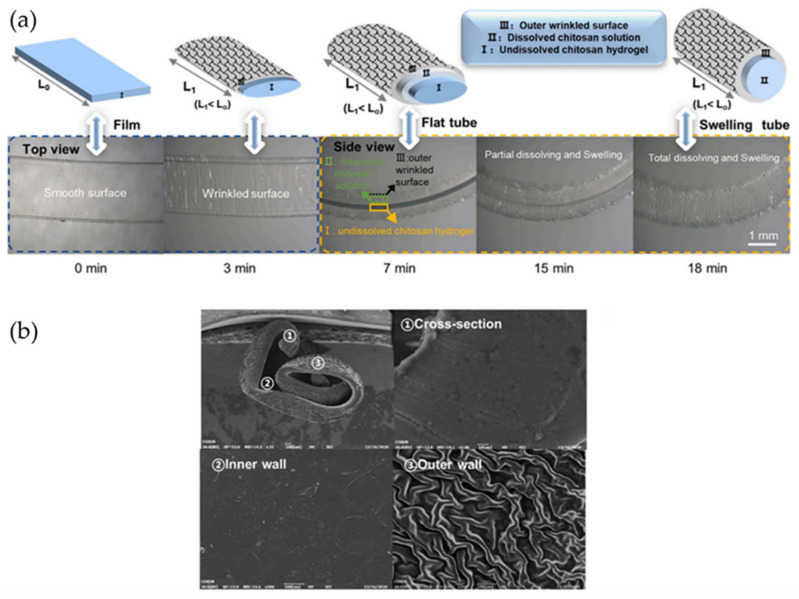
Wrinkled pattern tube formation process. (**a**) Microscopic and scheme of wrinkled tube device and (**b**) Cross-section morphology of the inner and outer wall of the wrinkled tube. Reproduced with permission from reference [123]. Copyright 2019, American Chemical Society (ACS).

Along with the procedures already presented, Shi et al. report a hollow chitosan hydrogel tube that was electrodeposited with controllable wavelength and wrinkle amplitude by immersing the film in acidic sodium dodecyl sulfate (SDS). A more in-depth analysis of this procedure establishes two main steps. Firstly, electrodeposition of chitosan-dissolved solution with a titanium plate via a cathodic neutralization mechanism. Secondly, an SDS soaking process generates the tubular configuration and the controllable surface wrinkles. In Figure 31, the group schematizes the methodology reported to facilitate an understanding of the process. Furthermore, this group reports that the resulting wrinkled hydrogel tube exhibits excellent anti-fatigue capacity, mechanical strength, flexibility, and resilience [123].

## 4. Complex Wrinkles Patterns

As we already mentioned in the previous sections, the formation of wrinkled microstructured patterns in the material’s surface has many advantages for some applications, mainly due to the increase of available contact area between the material and the environment, which could generate several improvements in the development of some particular and exciting devices like chemical/pressure [124,125] and piezoresistive sensors [126], among others. This section will be focused on reviewing the formation of complex wrinkled patterns like gradient and hierarchical patterns and some of their most relevant applications.

### 4.1. Strategies for Fabricated Gradient Wrinkle Surfaces

As demonstrated in the previous sections, the size and structuration of the wrinkled patterns could be controlled via the alteration of some particular variables like type of compression/relaxation (uniaxial, biaxial isotropic, biaxial anisotropic, etc.), stiffness or thickness of the rigid upper layer, among others. Uniaxial compression in the film results in striped wrinkles, but isotropic biaxial compression generates randomly oriented labyrinth-like wrinkle patterns or ordered herringbone patterns. However, the transition from disordered to ordered wrinkle patterns with particular orientation has been reported to occur from symmetry breaking caused by the anisotropy of compaction in their gradients. Accordingly, and with the finality of studying these symmetry-breaking processes, the fabrication of gradient wrinkled structures could be helpful. Indeed, Yu et al. [127] investigated and reported the wrinkling of patterns in tantalum (Ta) film sputters deposited on the modulus-gradient PDMS substrate. Due to the intrinsic and thermal stresses, wrinkling patterns spontaneously form on the sample surface. The wrinkles’ size strongly depends on the substrate stiffness and film thickness (decrease steadily increasing the substrate modulus but increase linearly with the thickness). The gradient sample’s hydrophobicity increases with the wrinkling pattern aspect ratio.

Similarly, Yu and Ni et al. [128] report a methodology to control the evolution of folds into wrinkles and create hierarchical wrinkles on the surface of a given soft material by regulating substrate stiffness and sputtering flux, producing a transition from lattice folds to isolated folds and wrinkles, which finally lead to labyrinth-like wrinkles. Furthermore, two distinct wrinkling patterns (G1 and G2) can coexist on the sample surface for a more considerable sputtering flux. Also, Yu and Ni et al. propose an unconventional methodology to form periodic thickness metal films on PDMS foils by masking copper meshes using room temperature DC magnetron sputtering. The thickness of the metal layer is directly related to the wrinkle structure generated; thus, the film surfaces at the mesh centers transition from labyrinth-like wrinkles to herringbone wrinkles and then to stripe-like wrinkles until a wrinkle-free state is reached when the mesh size is reduced or the film thickness is increased.

Likewise, Kim et al. [129] presented a simple process of fabricating a wrinkle pattern with a gradual gradient with a spatially controlled discrete wavelength in distinct zones with clear boundaries on a PDMS substrate. They performed a sequential oxygen plasma treatment for PDMS surfaces, employing a glass plate to prevent this perturbation partially. First, the PDMS substrate was fixed and stretched using a stretching device with 10% pre-strain in a uniaxial orientation. It generated a stable wrinkled groove structure without unexpected bifurcation of the wrinkled groove structure. Subsequently, the PDMS pre-stretched with the glass plate was exposed to oxygen plasma for 12 min. After the first exposure, the glass plate was gently removed to perform additional plasma treatment sequentially for 6 and 3 min, generating distinct regions with three discrete wavelengths. This research further studied sequential and continuous plasma treatment, finding discrepancies in values when the total time was 12 min.

**Figure 32:**
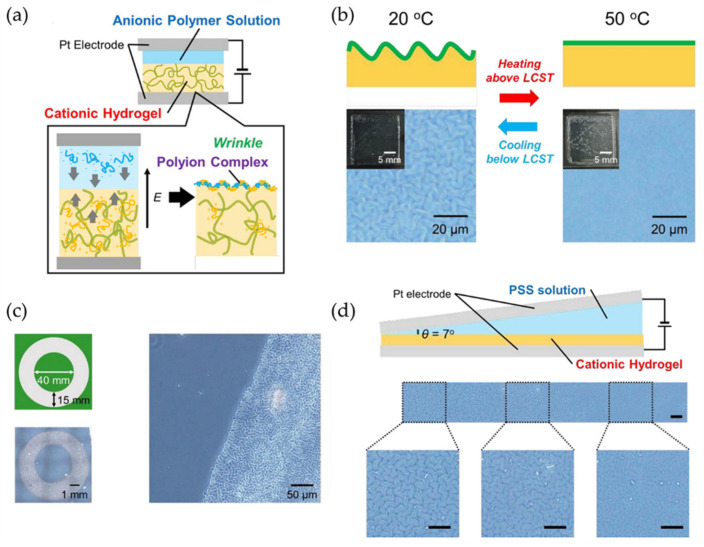
(**a**) Schematic description of the experimental procedure followed to fabricate wrinkled surfaces, (**b**) stimuli-responsive wrinkle structures at the surface of PNIPAAm hydrogel, (**c**) illustration of the Pt electrode’s insulting mask patterns, and (**d**) schematic representation of the experimental procedure of gradient wrinkle structure formation using sloped electrodes. Reproduced with permission from reference [130]. Copyright 2019, Royal Society of Chemistry (RSC).

On the other hand, Kato et al. [130] have developed a novel procedure to create wrinkled patterns on the surfaces of hydrogels under aqueous conditions by electrophoresis. A cationic hydrogel was prepared from N,N-dimethyl acrylamide with N,N-methylene bis(acrylamide) in the presence of poly(diallyl dimethylammonium chloride) (PDDA) and subjected to an anionic polymer solution based on sodium poly(styrene sulfonate) (PSS). By applying an electric current through the hydrogel system, it was possible to form a thin layer of polyion complex on top of the hydrogels by free-radical polymerization due to the electrostatic interaction between the cationic PDDA and the anionic PSS, thus generating wrinkled patterns on top of the material (Figure 32a). It was possible to prepare patterned structures (Figure 32c) and gradients (Figure 32d) by modulating the electrode position. The material also shows thermoresponsive characteristics; on the surface of the hydrogel, demonstrating a surface change from (wrinkled to flat) when varying the temperature (above or below the LCST, Figure 32b).

**Figure 33:**
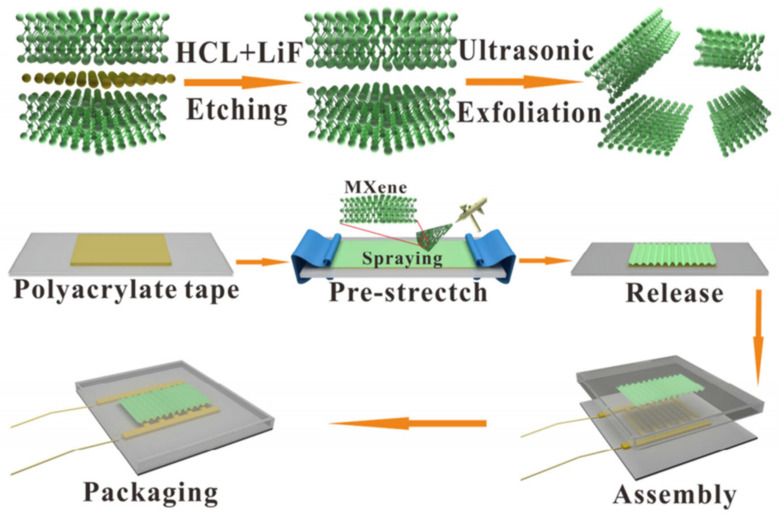
Schematic diagram of the preparation procedure used to fabricate the piezoresistive sensor based on MXene composite with wrinkled structure. Reproduced with permission from reference [126]. Copyright 2018, Elsevier Ltd.

Similarly, Liguori et al. [131] Differentiating stromal cells from adipose tissue was induced using wrinkled gradient models with nano- and microarchitectures on PDMS substrates. Cells were stimulated with TGF-β1 to elicit myogenic differentiation over seven days. Cell alignment occurred at topographies over 1.75 μm in width and 0.63 μm in breadth. Similarly, adhesion alignment increased on surfaces over 0.73 μm and 0.15 μm in width and breadth, respectively. These results make it possible to conclude that adhesion, alignment, and wrinkle differentiation are directly related to signals at the topographical level, suggesting that nanotopography may be an innovative tool to inhibit fibrosis. Similar results were reported by van Rijn et al. [132] but using mesenchymal stem cells to differentiate them into neural cells. Their results indicate that hierarchical structures significantly enhance neural differentiation, providing a new perspective in biomaterial development for tissue engineering applications. These gradient structures are advantageous in some areas, like flexible electronics and soft robotics. Indeed, Gao et al. [126] report the fabrication of an adjustable and highly-sensitive piezoresistive sensor based on 2D MXene material, which possesses wrinkled structures on its surface. The MXene was synthesized via wet chemical techniques; first, LiF was added in an HCl solution, then Ti_3_AlC_2_ powder was gradually added to the solution, and it was etched at 35° for 24 h, obtaining a dark green supernatant of Ti_3_C_2_T_x_ (MXene) with a pH over 6. The sensing device was fabricated by spraying MXene on a pre-stretched polyacrylate tape (50%, 100%, and 150% strain). After releasing the pre-stretched polyacrylate tape coated with MXene, the composite sensing element with a wrinkled structure was fabricated. Figure 33 shows a schematic description of the process used for MXene device fabrication. The MXene composite-based sensor demonstrates high sensitivity, wide pressure range, short response time, and excellent durability; it has been possible to sense physiological signals, monitor robot postures, and spatially map pressure distributions.

Similarly, the group of Wang et al. [133] fabricates flexible and wearable piezoelectric pressure sensors based on single-walled carbon nanotubes (SWCNT), graphite flakes (GF), and thermoplastic polyurethane (TPU). The wrinkled structures were formed via pre-stretching of the TPU, which was previously exposed to carbon deposition. The material’s wavy structure provided high conductivity, lower detection limit, high sensitivity, good flexibility, and short-time response. The pressure sensor was successfully adapted to monitor human activities, like walking, jumping, and pulse measuring. Furthermore, a sensor array is assembled to map an object’s weight and shape, indicating its various potential applications, including human-machine interactions, health monitoring, and other wearable electronics.

**Figure 34:**
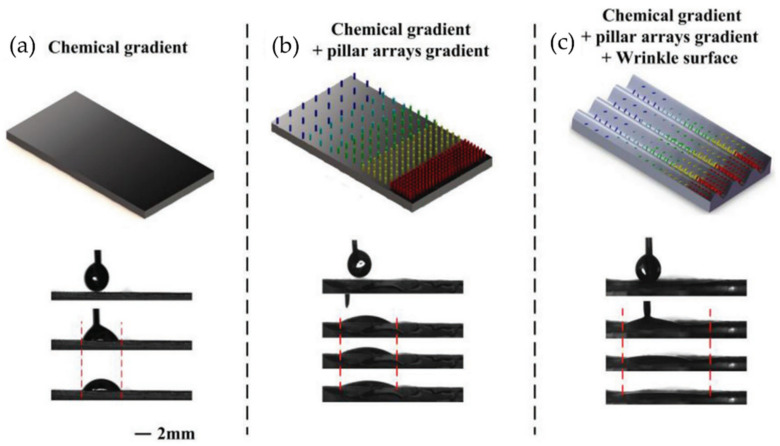
Droplet movement behavior on the surfaces with (**a**) chemical gradient, (**b**) chemical gradient and pillar arrays gradient, and (**c**) double-gradient wrinkled structure. Reproduced with permission from reference [134]. Copyright 2020, John Wiley & Sons, Inc.

Chai et al. [134] also developed a methodology to fabricate surfaces with controlled wettability through a series of surface material modifications (chemical gradient, pilar array arrangement, and wrinkled surface) (Figure 34). The chemical gradient is induced via oxygen plasma non-planar exposure over the PDMS substrate. In general, it was possible to demonstrate that the pilar density increases the wettability of the sample. Chai et al. proposed that the spreading length of droplets is regulated in situ by applying external strain.

Recently, Park et al. presented a strategy with a high level of security against imitation, using wrinkled patterns with various complexities through the development of non-clonable codes. This group fabricated wrinkled gradient patterns utilizing grayscale lithography with a custom mask with different gray levels, generating a controlled photopolymerization of a microparticle/silica-coated substrate. Even though, in most cases, the material implemented in the wrinkle formation has the major part in the matter, these authors propose a gray mask design depending on the factor. This custom gradient gray mask was designed to allow 15 gray levels within the same microparticle mask. It should be noted that the synthesized particles used different wavelengths for each gray level, establishing a total of 21 possible gray levels, each with eight gray level masking units, allowing a controlled gradient wrinkled pattern to be generated [135].

### 4.2. Hierarchically Ordered Wrinkled Surfaces

In the same way as wrinkled gradient topographies, hierarchical wrinkled patterns could also be generated under particular circumstances. The group of Genzer et al. [29] reports a multi-step procedure that was employed to generate hierarchical crumpled patterns. With this new method, they could form self-similar strands extending over five orders of magnitude on the length scale, ranging from nanometers to millimeters. Recently, Odom et al. [136] transformed flat polystyrene (PS) plates into three-dimensional hierarchical structures by using sequential cycles of mechanical stretching and trifluoromethane (CHF_3_) plasma exposure, followed by a stress relaxation process. Several studies were also carried out to identify the main aspects that dominate hierarchical wrinkled patterns’ formation and their topology distribution. For example, Begging and Xu et al. [137] prepared wrinkled surfaces using lithography processes over soft PDMS substrates. SU-8 pillars grown on silicon wafers were used to create holes in the substrate via the spin coating method. The PDMS substrate was then transferred to a soft pre-stretched layer and released to form parallel wrinkled patterns over the surface. The evolution of parameters was studied, like distribution, size and separation of holes, and strain percentage (6%, 15%, 30%, and 55%).

On the other hand, Hu et al. [138] demonstrate that controllable hierarchical surface patterns can be fabricated by combining nano embossing techniques and surface instability of supramolecular hydrogel. Their article used polyethylene glycol (PEG) based hydrogels to fabricate nanostripe arrays upon water exposure. Due to the lateral expansion of the stripes, folded patterns were formed on the material’s top. This way, hierarchical structures could be easily assembled, one due to the nano embossing process to create the nanostripes and the other by the surface buckling. The dimensions of the patterns could be controlled via the nanostripe pattern dimensions and by the swelling time.

**Figure 35:**
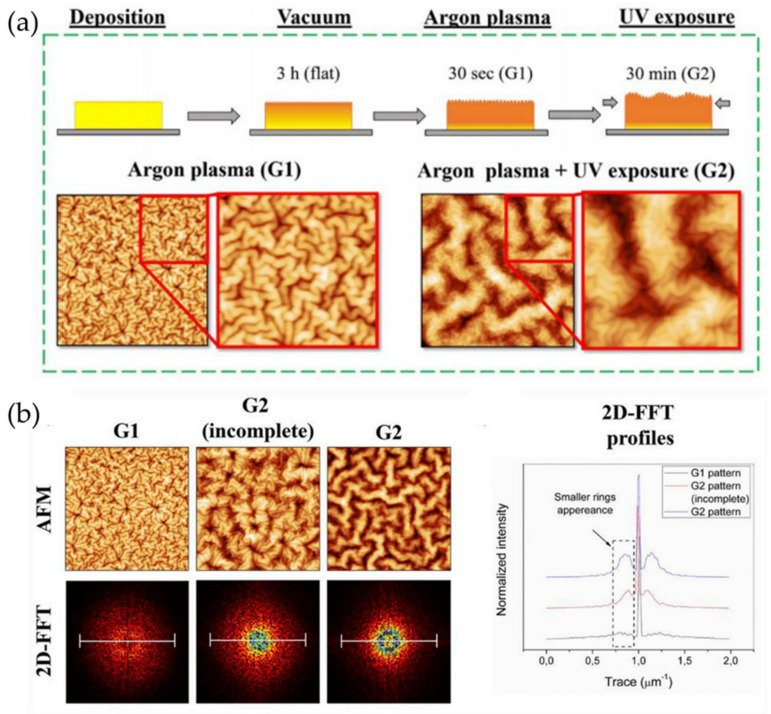
(**a**) Schematic illustration of the methodology used to fabricate hierarchically structured wrinkled hydrogel films, together with AFM micrographs for each sample 15:1 net-poly(DMAEMA-*co*-PEGDA_575_) and (**b**) AFM micrographs of G1, incomplete G2 and fully formed G2 wrinkled patterns, 2D-FFT profile are depicted right. Reproduced with permission from reference [139]. Copyright 2019, Elsevier Ltd.

Likewise, the González Henríquez et al. [139] have proposed a multi-step strategy to form flat, first and second-generation structures using vacuum, argon plasma, and UV exposure steps. To achieve these wrinkle dimensions, different concentrations of monomers such as 2-(dimethylamino)ethyl methacrylate (DMAEMA) and poly(ethylene glycol)diacrylate (PEGDA_575_) were mixed. Besides, Irgacure 2959 was used as a photo-initiator, ammonium peroxodisulfate (APS) as a thermo-initiator, and sodium metabisulfite (SPS) as a catalyst. The APS-SPS was employed as redox pair with the finality to initiate the gelation reaction. To create G1 and G2 patterns, three essential steps must be performed. First, water removal via vacuum generates a gradient in the material, inducing G1 patterns. Second, frontal vitrification is caused by argon plasma exposure, triggering the formation of G2 wrinkles at the top of the G1 pattern. Thirdly, exposition to UV to completely polymerize the surface and fix the pattern to the substrate (Figure 35a). Figure 35b shows three AFM images corresponding to a G1 pattern, an incomplete G2 pattern (lower UV exposition time), and a G2 fully formed.

Recently, Choi et al. [140] also developed a simple method to fabricate multiscale wrinkled patterns with two different structure generations (G1–G2) using fluorocarbon thin films over the PDMS substrate. The elastic properties of the substrate greatly influenced the wrinkled structures when subjected to the fluorocarbon polymer sputtering process; in fact, it is quite possible that some of the wrinkled structures were formed in this time span.

Several of these studies were also performed using metal films to elucidate the evolution of the hierarchical wrinkled morphologies related to several external stimuli. For example, Zhang et al. [141] reported creating hierarchical wrinkles and oscillating cracks in metallic films deposited on scratched silicone oil (or PDMS). Thus, the results demonstrated that wrinkled patterns strongly depend on the stripe width and film thickness. Generally, straight wrinkles were formed near the stripe edge, while labyrinth-like wrinkles appear at the center.

Additionally, as the distance from the edge increases, the wrinkle wavelength increases quickly, and the growth speed slows down gradually. Three distinct wrinkles were observed in the cracked films: hierarchical, wavy (or herringbone), and straight structure. The formation of complex wrinkles in cracked films is attributed to the constrained (or free) edge effect and stress anisotropy. Thus, the wavelength of both hierarchical and wavy wrinkles increases with film thickness.

Another study that elucidates thin metal films’ behavior against compressive/expansion stimulus was carried out by Jiao and Yu et al. [142]. It reported spontaneous hierarchical wrinkling in metallic films deposited by sputtering on a liquid gel substrate. Molybdenum (Mo) metal films (from 1.3 nm to 600 nm) were deposited on the non-crosslinked gel substrate (~18 μm thickness PDMS) by magnetron sputtering technique. In this publication, metal atoms and clusters can penetrate the gel substrate in the initial phase of sputtering, and complex surface structures are formed during deposition. The compressive pressure generated by the volume expansion forces the gel surface to generate highly deformed ridge structures, referred to as wrinkles. Thus, the wrinkles have a disordered appearance caused by the isotropic stress field. Also, the wavelength of the wrinkles decreases steadily with increasing separation but is insufficiently sensitive to film thickness relative to the prediction of the elastic theory. The differences in size and length between the spontaneously forming hierarchical structures should benefit various applications, be they photoelectronic devices, surface engineering, and biology-related fields.

There are several applications in which these patterns could be used; for example, structures with hierarchical roughness on both the nanoscale and microscale could help to improve superhydrophobicity. [143]. Indeed, Carter et al. fabricate nanoscale patterns on poly(hydroxyethyl methacrylate) (PHEMA) film by nanoimprint lithography via a reactive methylchlorosilane infusion reaction. The morphology and size of the surface structure are easily controlled by adjusting the initial thickness of the PHEMA films.

Unlike randomly distributed wrinkles, hierarchical wrinkles exhibit interesting orientations, mainly due to pre-imprinted patterns and confinement effects. In general, hierarchically wrinkled surfaces have significantly higher water contact angles than random wrinkled surfaces, showing in some cases superhydrophobicity behavior (water contact angles higher than 160° and sliding angles lower than 5°).

Wu and Chai et al. [144] report a theoretical framework that describes how prestrain and dynamic strain could reversibly tune the wettability of hierarchically wrinkled surfaces. Commercial silicon templates were used to fabricate hierarchical wrinkled surfaces. Then, a PDMS solution was deposited on the material via spin-coating and thermally cured. Finally, a VHB-prestrained film was adhered to the PDMS and heated together to create a strong bond between the films. The system was slowly cooled and released to form wrinkled structures, creating a hierarchical pattern of nano and micro wrinkles on the material. The wetting properties of the material varied with the dimensions and ordering of the wrinkles. It was possible to demonstrate that the influence of prestretch and applied stretching affects wettability. By analyzing the experimental and theoretical results, a relationship between the contact angle and structural size using a thermodynamic equation was established. Furthermore, the surface wettability can be adjusted from superhydrophobic to hydrophobic, even after multiple stretching-release cycles.

Similarly, Yin et al. [145] fabricate smart windows with a variable hydrophobic performance using dynamically tunable hierarchical wrinkles. These devices should present water droplet transport control using self-similar hierarchical wrinkles over PDMS substrate through sequential strain release cycles. They demonstrate that the re-stretching/releasing of the elastomeric material leads to a reversible and repeatable switching between opaqueness and transparency. Yin et al. also show the capacity of the material to modify its water droplet transport; initially, when a water droplet is deposited on the surface, the droplet stays pinned due to the hierarchical pattern presence, then the droplet starts to slide when the surface is stretched and becomes pinned again upon strain release. The sliding process occurs because first-generation wrinkles loosen due to material stretching, leading to a simple wrinkled surface pattern. When the substrate is released, the second-generation wrinkled hierarchical pattern emerges again, thus pinning the drop.

These hierarchical wrinkled patterns could also serve as a base for fabricating pressure sensors; for example, in 2018, Lu et al. [146] manufactured highly-sensitive pressure sensors based on polypyrrole (PPy) films, which are composed of three-scale nested surface wrinkling microstructures. Double-scale nested wrinkles are formed via spontaneous wrinkling after oxidative polymerization growth. The polymerization time is fundamental for this process, allowing the pattern’s dimensions to be modulated easily. Heating and cooling cycles induce the third generation of surface wrinkling. The dimensions of the third-generation wrinkles are directly related to the heating temperature and heating cycle duration. These multiscale hierarchical structures, combined with the materials’ stimulus-responsive characteristics, allow the fabrication of pressure sensors with high sensitivity, low detection limit, ultrafast response, excellent durability, and time stability, being suitable as an integrative part of wearable electronics.

Accordingly, hierarchical wrinkled surfaces could also be used for different applications in the wearable electronics field; for example, Zhang and Wang et al. [147] proposed a method to fabricate wearable triboelectric nanogenerator (TENGs) based on hydrogel wrinkled electrospun nanofibers. These hierarchical structures form a membrane for effective energy harvesting from human movement. The PDMS substrate was packaged with PAAm-LiCl hydrogel (polyacrylamide hydrogel containing lithium chloride) to fabricate the TENG. A copper belt is embedded inside the hydrogel as an electronic connection. An air layer is added to the middle of the devices as a contact separator; air can enter and leave freely through holes in the walls on both sides of the devices under regular operation. Posteriorly, a polycaprolactam (PA6) solution was deposited via electrospinning onto the PAAm hydrogel’s surface. Finally, the films are released by cooling the flask to obtain a hierarchically wrinkled PA6 membrane. This device presents high stretchability and enlarged triboelectric area, improving its performance due to the formation of rough surfaces. These outstanding features may optimize TENGs devices for fitness braces.

Following the topic of flexible electronics, Cui and Liu et al. [148] fabricated a hierarchical Cu(OH)_2_/MnO_2_ core-shell nanorod array on copper foam (CF), utilizing Cu(OH)_2_ nanorods in situ generated on the Cu foam. Thus, the Cu(OH)_2_/MnO_2_/CF nanorods array display an excellent capacitance of ~709 mF cm^−2^ at the current density of 2 mA cm^−2^ (~283 F g^−1^ at 0.8 A g^−1^), favorable rate capability (74.65%, from 1 to 20 mA cm^−2^) and great cycling stability (85.17% of capacity retention after a test for 5000 cycles). Additionally, the assembled Cu(OH)_2_/MnO_2_/CF/activated carbon (AC) asymmetric supercapacitor shows an outstanding energy density of ~18 Wh kg^−1^ at a power density of 750 W kg^−1^, and excellent cycling stability with 90.03% capacitance retention after 6000 cycles.

**Figure 36:**
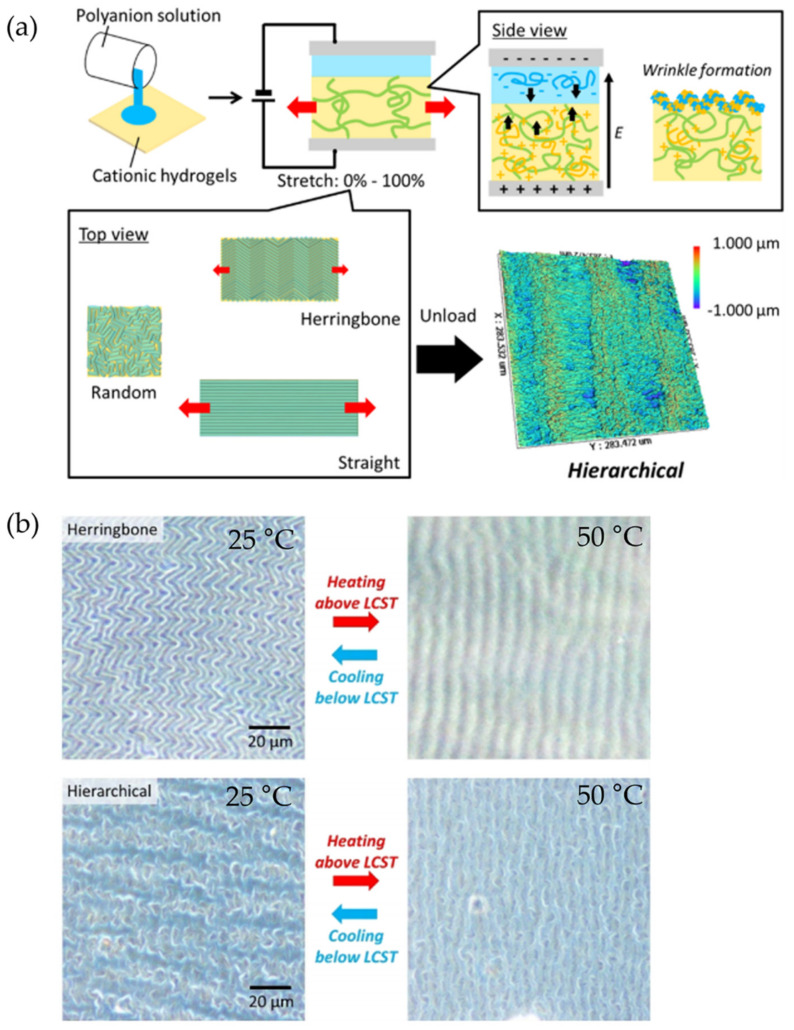
(**a**) Experimental procedure for wrinkle formation on the hydrogel surface by electrophoretic fabrication under stretching and (**b**) thermoresponsive transformation of herringbone and hierarchical wrinkles structures. Reproduced with permission from reference [149]. Copyright 2020, American Chemical Society (ACS).

On the other hand, wrinkled hierarchical structures could also respond to an external thermal stimulus; for example, Asoh et al. [149] studied aligned wrinkles fabricated via electrophoretic polymerization of a polyion complex (PIC) as a hard-skin layer. The cationic PDMAAm hydrogels with semi-interpenetrated network structures were prepared by free-radical polymerization of N,N-Dimethylacrylamide (DMAAm) with methylenebisacrylamide (MBAAm). The wrinkles on the hydrogel surface were prepared under stretching; aqueous sodium poly(4-styrene sulfonate) (PSS) solution was poured on the top of a stretched cationic hydrogel and then sandwiched between two pieces of Pt electrodes. The stretching ratio of hydrogels during the electrophoresis was changed to control the wrinkle formation. An aligned wrinkled structure was fabricated on the surface of oriented hydrogel using poly(vinyl alcohol) (PVA) and PPA dissolved in water. Then, 25% glutaraldehyde (GA) solution as the cross-linker and hydrochloric acid as the catalyst were added to the solution. Wrinkle formation was performed on the surface of oriented hydrogel, and the applied electric fixed was fixed to 10 V mm^−1^. The wrinkle geometry was controlled by modifying the electrophoretic conditions and Young’s modulus of the hydrogels. Figure 36a indicates the topological modulation of hydrogel wrinkling as a function of stimuli based on the fabrication of aligned and hierarchical wrinkle structures in the hydrogel. The stretching ratio, Young’s modulus of hydrogels, and hierarchical wrinkle structures were fabricated after releasing the stretched hydrogels (Figure 36b).

**Figure 37:**
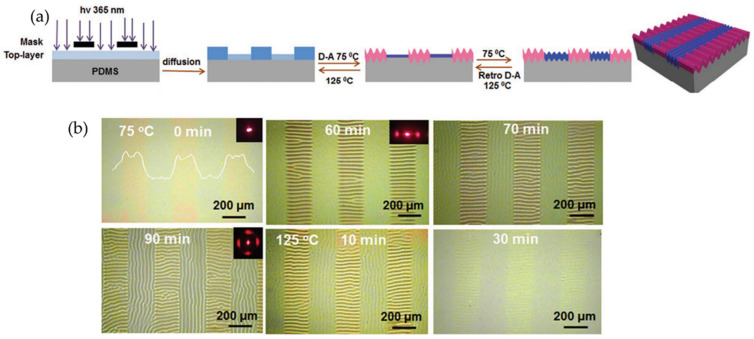
(**a**) Schematic description of the process used for fabricating hierarchical patterns with dynamic wrinkles through a photo-controlled Diels-Alder reaction and (**b**) optical images of the formation/extinction process of horizontally or vertically striped wrinkles on reversible Diels-Alder reaction. Reproduced with permission from [150]. Copyright 2020, John Wiley & Sons, Inc.

Recently Jiang et al. [150] proposed a simple but robust method to fabricate 3D patterned surfaces with dynamic topographies by taking advantage of the reversible DA reaction. In this article, Jiang et al. synthesize a substrate composed of a copolymer containing furan (PSFB) and bismaleimide (BMI), which can selectively polymerize through photodimerization of BMI by a mask when exposed to UV light (Figure 37a). The spread of maleimide from the unexposed region to the exposed region results in a diffuse relief pattern. By controlling the reversible D-A reaction at different temperatures, orthogonal wrinkles can be sequentially and reversibly generated or erased in the exposed and unexposed regions of the surface, thus fabricating dynamic wrinkle patterns that actively respond to ambient temperature (Figure 37b).

Some applications also involve the use of light-reversible hierarchical wrinkled patterns, like the case of Jiang et al. [151]. A straightforward and efficient strategy has been created to fabricate light-manageable hierarchical patterns with highly reversible morphology and fast turnaround time through dynamic reversible wrinkle-induced dynamic photodimerization.

**Figure 38:**
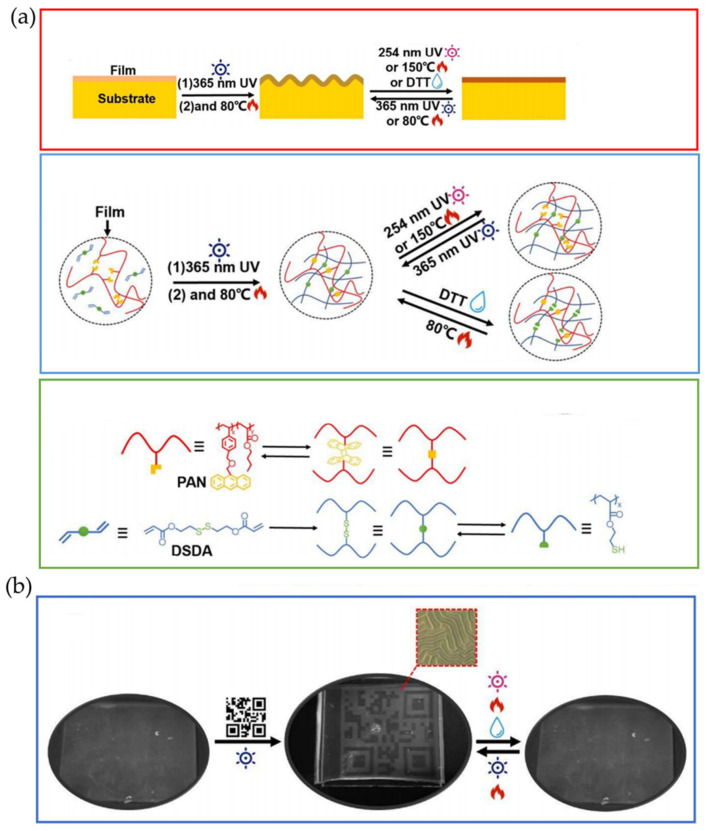
(**a**) Schematic diagram of the IPN fabrication and transformation process and the corresponding wrinkle generation/quenching process. Bilayer wrinkle generation/quenching process (red). IPN formation/quenching in the film by multi-stimuli (blue). The reversible chemical reaction of PAN and DSDA in the skin layer (green). (**b**) Photography of QR code with the reversible wrinkled pattern. This pattern was fabricated using UV irradiation of 365 nm and erased using 254 nm in the UV range. Reproduced with permission from reference [152]. Copyright 2019, American Chemical Society (ACS).

Jiang et al. reported obtaining wrinkles that deliver a multi-response and surface geometry that can be formed as well as dynamically removed in situ by multiple physical or chemical stimuli, such as UV and an aqueous solution of DL-1,4-dithiothreitol (DTT, reductant) and heat (Figure 38a). The wrinkles were formed by the dynamic cross-linking of the interpenetrating polymeric network (IPN) containing covalent bonds sensitive to redox-type reactions and visible light, designed as the top layer deposited on PDMS elastomers. Given the dynamic reversible covalent -C-C- bond in the anthracene-containing polymer (PAN) and -S-S- bond in the disulfide-containing diacrylate monomer (DSDA), the surface morphology can not only be reversibly and precisely adapted to all kinds of complicated hierarchical patterns permanently, but it can also be controlled by NIR radiation. The authors fabricated a QR code with wrinkle patterns, which is visible to the naked eye due to light scattering from the wrinkles (Figure 38b).

**Figure 39:**
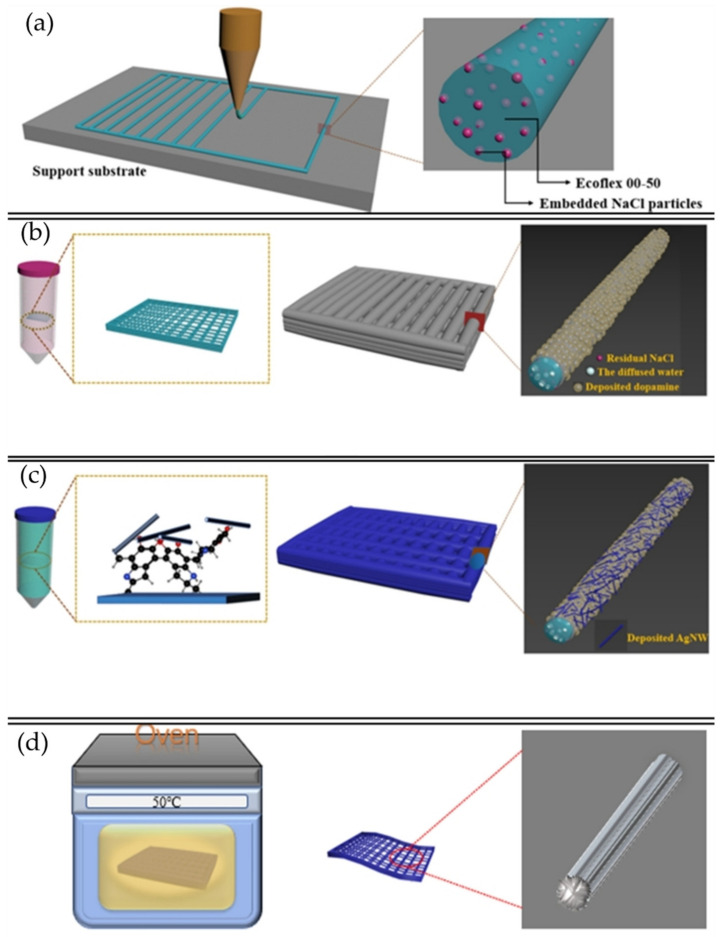
Schematic description of the fabrication process of stretchable elastomer sensors. (**a**) Formation of structures by 3D printing. (**b**) Dopamine treatment via immersion in an aqueous solution. (**c**) Deposition of AgNW on the dopamine-coated sample and (**d**) formation of rough structures after remotion of residual water. Reproduced with permission from reference [153]. Copyright 2022, Elsevier Ltd.

Finally, in 2022, Yang et al. implemented a swelling base wrinkled structure manufacturing process. This group generated stretchable sensors from silver nanowires (AgNW) that exhibit a crumpled hierarchical structure. These structures were fabricated through a two-step process: (1) water-induced swelling and (2) AgNW soaking in a dopamine aqueous solution. It is worth mentioning that the swelling stage endows the sample surface with ultra-hydrophilicity. Subsequently, the dopamine-modified swollen samples fixate the nanowires when immersed in the AgNW/ethanol solution due to the dopamine’s highly adhesive layer properties. In Figure 39, it is possible to observe a schematic description of the fabrication of these personalized stretchable elastomer sensors using 3D printing [153].

## 5. Conclusions and Future Perspectives

The microstructure and chemical composition of polymer surfaces are critical aspects that directly or indirectly control the material characteristics and how it behaves with its surroundings; therefore, it influences the final application of the material.

This review mainly focused on wrinkle formation on polymer surfaces using smart materials that can react to slight environmental changes. This type of structuration is obtained by taking advantage of the surface instabilities generated, which result in an out-of-plane deformation. This process can be controlled; thus, the final wrinkle characteristics, including period and amplitude, can be finely modulated.

More recently, a great effort has been focused on elaborating more sophisticated surface structures, including (1) reversible stimuli-responsive wrinkle formation, (2) non-planar wrinkled structures, and (3) complex wrinkled patterns like gradient or hierarchical surfaces. These strategies have also been summarized and organized in different sections in the present review providing a broad overview of the current possibilities to modulate the interfacial properties.

Firstly, reversible stimuli-responsive wrinkled patterns were formed using smart polymers that can respond to external stimuli and actively react to environmental changes. In this section, materials that can react to stimuli were reviewed, the most common being light stimuli-responsive materials such as the one reported by Omenneto et al. [40] or Jiang et al. [43]; also, changes in environmental pH were reviewed, as in the case of Yang et al. [59], or temperature stimuli-responsive materials as in the case of Zhang and Sun et al. [60]. Other types of stimuli were also reviewed, such as changes in electric/magnetic fields or changes in environmental humidity and even reactions to mechanical stimuli such as stretching or compression. In addition, a series of applications, such as logic gates or anti-counterfeiting devices, were also mentioned and analyzed.

Secondly, non-planar wrinkled structures were reviewed, standing out several structures like solid core-shell spheres or cylinders and hollow spheres or cylinders. Interestingly, these types of structures allow taking more advantage of the surface increment that generates the wrinkling process by creating these patterns on non-planar surfaces, thus improving several characteristics that depend on the material’s interaction with the environment. One of the most exciting applications is, for example, the use of core-shell or hollow spheres for drug delivery or the use of cylindrical hollow tubes that can react to temperature or NIR stimulus, thus allowing to open or close a channel depending on the external stimulus (Sun and Wu et al. [122]).

Finally, complex wrinkled patterns were also reviewed in this article, mainly focused on the formation of gradient and hierarchically ordered wrinkled patterns. The fabrication methods for this type of complex structure go from taking advantage of surface instabilities using UV, plasma, oxidation, or mechanical stretching, to more advanced fabrication methods like electrophoresis, as reported by Kato et al. [130]. These complex wrinkled patterns, together with some particular functionalities of the smart materials, allow the development of incredible applications like anti-counterfeiting devices or surfaces with controlled wettability (Chai et al. [134]).

The developments in this review about wrinkle structure formation may provide an exciting toolbox to produce surfaces adapted for a particular purpose and able to change their structure and, thus, their properties depending on the environmental conditions.

## Figures and Tables

**Table 1:** Summary of light-responsive materials listed in Section 2.1.

Base Material	Responsive Light Range	Wrinkling Methodology	Posible Application	Ref.
Pyridine-containing azopolymer	Visible light	- Exposure to high-intensity white light - Selective exposure with a mask - Oxygen plasma exposure	Rewritable data storage	[41,42,43]
Pyridine-containing polymer		- Thermal treatment at 110 °C - Compressive stress - Light exposure	Anti-counterfeiting	[44]
Epoxy-based azobenzene		- Heating to 90 °C for 1 h - Cooling to RT - Light exposure	Rewritable data storage	[46]
(POD-PS)/PDMS		- 450 nm laser exposure - Cooling to RT - Selective exposure with a mask	Rewritable data storage Anti-counterfeiting	[54]
PET/LAP	UV light	- Heating to 90 °C for 1 h - Cooling to RT - Light exposure	Photonic devices Cell culture patterns Flexible sensors	[49,50]
Silk/PDMS		- Heating at 140 °C (5 min) - Cooled to RT - UV Light exposure	Rewritable data storage Anti-counterfeiting	[40]
CNT/PDMS/PAN	NIR light	- Heating to 70 °C - Cooling to RT - NIR exposure	Anti-counterfeiting	[51,52,53]
CNT/PDMS		- Heating to 80 °C for 3 min - Cooling to RT - UV light exposure for 40 min	Smart displays Dynamic gratings Light control electronic	[47,48]

**Table 2:** Summary of pH-sensitive materials listed in Section 2.2.

Base Material	Responsive pH Range	Wrinkling Methodology	Possible Application	Ref.
PAA/PEG/PDMS	2.5–7.0	- Heating at 200 °C for 1 h - Immersion in acidic solution pH 2.5	Tunable stress-relief patterns	[58]
PAA/PAH/SiO_2_	4.5–6.5	- Immersion in acidic solution pH 2.0 - Immersion in pH 5.5 solution for 5 min - Exposure to 40% RH environment	Morphology-controlled smart superhydrophobic films	[57]
PNIPAM-co-AA/CuS	5.6–9.2	- Deposition of CuS - Freeze-drying for 1 day	Biomimetic mineralization	[59]

**Table 3:** Summary of thermo-sensitive materials listed in Section 2.3.

Base Material	Responsive Temperature Range	Wrinkling Methodology	Possible Application	Ref.
PNIPAM and hybrid silica	RT—36°C	- Sample drying for 15 min - Sample stretching/releasing - Hybrid silica deposition - Heating to 36 °C	Soft micro-actuators	[61]
PEGDMA/tBA	RT—60 °C	- Sample stretching/releasing (5% strain) at 60 °C. - Cooling to RT - Aluminum film deposition	Smart micromirror	[65]
PVA/PDMS	RT—80°C	- Sample stretching/releasing (15% strain) - Exposure to moisture (70% RH) or high temperature (80 °C)	Smart windows	[63,64]
PS/PEM/CL	RT—100 °C	- PS argon plasma exposure - Heating to 100 °C - Cooling to RT - Deposition of CL layer	Fuel cells	[66]

**Table 4:** Summary of mechanical (stress/stretch) listed in Section 2.5.

Base Material	Responsive Range	Methodology	Application	Ref.
PVA/PDMS	Stretching	- Oxygen plasma exposure - PVA immersion on PMDS - Drying at RT - Stretching	Dynamic optical switching	[71]
PDMS	Stretching	- PMDS pre-stretch - Mask coating - Vacuum exposure (15 min) - Stress release	Mechanochromic	[72]
PVP/Ir/PDMS	Stretching	- Stretching/relaxation	Structural health monitoring Soft robotics	[73]
PDMS	Stretching	- Stretching (200%) - UVO exposure (30 min) - Stress removal	Portable electronics	[74]
PPY/PDMS	Pressure	- PPY film deposition on PDMS - Phase oxidation polymerization	Optically switchable microcircuit and photodetection	[75]
SU8/PDMS	Stretching	- -Pre-stretch - Deposit SU8 film in PMDS - System compression - Gold film deposition - Reversible compression	Advanced flexible electronics	[76]
PDMS	Stretching	- Film stretching (16%) - Air plasma exposure (5 min) - Relaxation (30 s)	Microfluidics	[78]

## Data Availability

Not applicable.
